# An Overview and Evaluation of Recent Machine Learning Imputation Methods Using Cardiac Imaging Data

**DOI:** 10.3390/data2010008

**Published:** 2017-01-25

**Authors:** Yuzhe Liu, Vanathi Gopalakrishnan

**Affiliations:** 1 Department of Biomedical Informatics, University of Pittsburgh, Pittsburgh, PA 15260, USA; 2 Medical Scientist Training Program, University of Pittsburgh, Pittsburgh, PA 15260, USA; 3 Department of Computational and Systems Biology, University of Pittsburgh, Pittsburgh, PA 15260, USA; 4 Intelligent Systems Program, University of Pittsburgh, Pittsburgh, PA 15260, USA

**Keywords:** missing value imputation, machine learning, decision tree imputation, k-nearest neighbors imputation, self-organizing map imputation

## Abstract

Many clinical research datasets have a large percentage of missing values that directly impacts their usefulness in yielding high accuracy classifiers when used for training in supervised machine learning. While missing value imputation methods have been shown to work well with smaller percentages of missing values, their ability to impute sparse clinical research data can be problem specific. We previously attempted to learn quantitative guidelines for ordering cardiac magnetic resonance imaging during the evaluation for pediatric cardiomyopathy, but missing data significantly reduced our usable sample size. In this work, we sought to determine if increasing the usable sample size through imputation would allow us to learn better guidelines. We first review several machine learning methods for estimating missing data. Then, we apply four popular methods (mean imputation, decision tree, k-nearest neighbors, and self-organizing maps) to a clinical research dataset of pediatric patients undergoing evaluation for cardiomyopathy. Using Bayesian Rule Learning (BRL) to learn ruleset models, we compared the performance of imputation-augmented models versus unaugmented models. We found that all four imputation-augmented models performed similarly to unaugmented models. While imputation did not improve performance, it did provide evidence for the robustness of our learned models.

## 1. Introduction

In biomedical research, samples with missing values are typically discarded to obtain a complete dataset. This method, known as listwise deletion or complete case analysis, reduces the sample size available for analysis and may bias the results [1,2]. Many statistical methods have been proposed to impute missing values, including last value carried forward, mean imputation, expectation maximization, and multiple imputation.

Since the early 2000s, a new paradigm of thinking has emerged where missing values are treated as unknown values to be learned through a machine learning model. In this framework, data samples with observed values for a particular variable are used as a training set for a machine learning model, which is then applied to the data samples with missing values to impute them. Both clustering (unsupervised) and classification (supervised) algorithms can be adapted for imputation.

In our previous work on the evaluation of pediatric patients for cardiomyopathy, we attempted to learn quantitative clinical guidelines for ordering cardiac magnetic resonance imaging (MRI) based on quantitative metrics from previous echocardiography [3]. Current guidelines indicate obtaining a cardiac MRI if echocardiography is inconclusive in the evaluation of cardiomyopathies. As this guideline is highly dependent on physician expertise, we sought to identify objective, quantitative markers on echocardiography that would be predictive of a subsequent positive cardiac MRI. We chose to predict positive cardiac MRIs as our classification task because they have prognostic as well as diagnostic value beyond echocardiography [4]. An accurate prediction of a subsequent positive MRI provides evidence for ordering the follow-up MRI, while an accurate prediction of a non-positive MRI can justify avoiding the scan. Our goal is to provide physicians with quantitative evidence for whether a follow-up cardiac MRI is likely to be useful.

**Figure 1:**
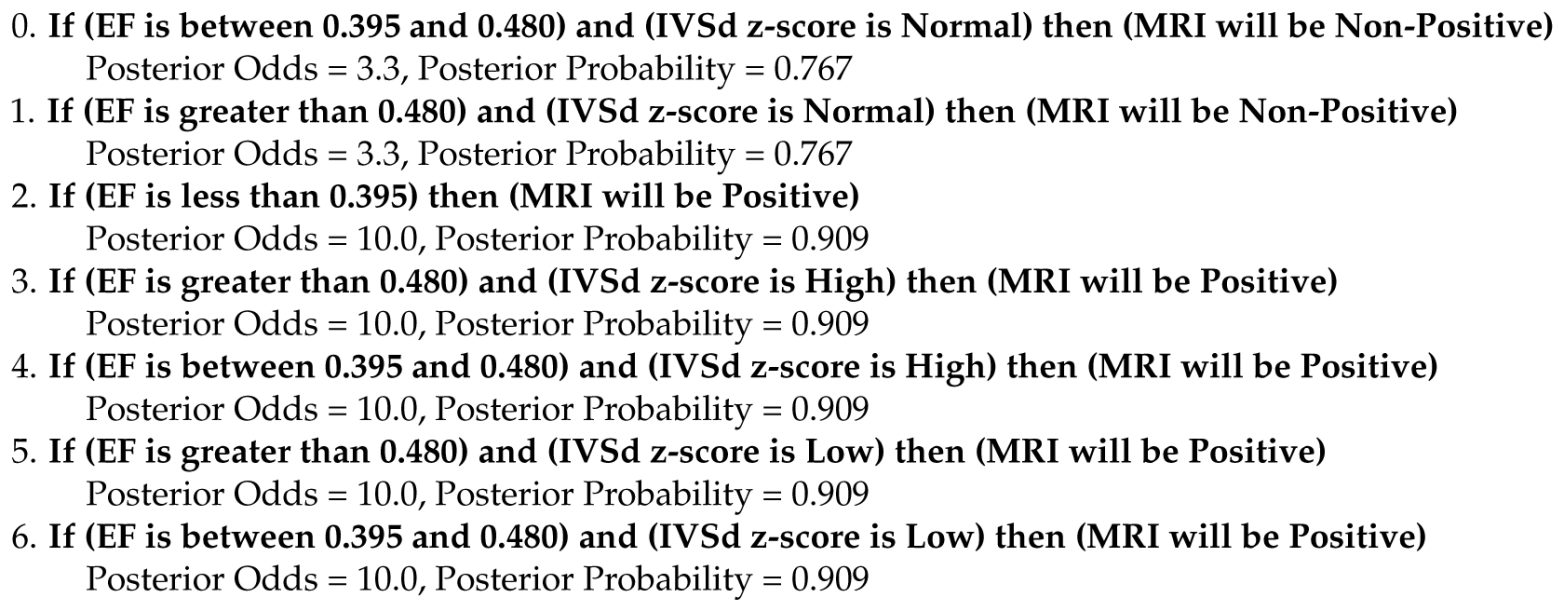
Example ruleset generated using Bayesian Rule Learning (BRL).

We obtained a dataset of echocardiographic measurements and follow-up cardiac MRIs from 88 pediatric patients undergoing evaluation for cardiomyopathy. This dataset contained missing values because not all echocardiographic measurements were recorded after an exam (see Table 1 for more details). After removing variables with a large number of missing values followed by listwise deletion to remove the rest of the missing values, we were left with 14 variables and 50 patients for our analysis. We used Bayesian Rule Learning (BRL), a variant of the Rule Learning algorithm that outputs probabilistic scores for each rule learned [5], to generate a ruleset that was predictive of a subsequent positive cardiac MRI. BRL generates a set of rules, an example of which can be seen in Figure 1. BRL performs better than logistic regression and on par with methods like SVM and decision trees on our data, and offers the advantages of human-readable rules with probabilistic confidence scores for each rule. The confidence scores make BRL especially suitable for small datasets like ours where some learned rules may have weak evidence.

Using BRL on our data after removing variables and samples with missing values, our learned rulesets on the 50 patients achieved around 90% specificity and 50% sensitivity in 10-fold cross-validation. In this paper, we sought to test two hypotheses: One, does increasing our dataset’s usable sample size through missing value imputation increase model performance? Two, does increasing our dataset’s usable sample size as well as variable count through imputation increase model performance? Finally, we sought to answer the question: When and why is imputation worth doing?

In Section 2, we first provide a brief review and summarize the current literature on machine learning methods for missing value imputation. In Section 3, we then evaluate the impact of several imputation methods on the performance of our previously described rule learning task.

## 2. Background: Review of Current Machine Learning Imputation Methods

### 2.1. Introduction and the Nature of Missingness

When imputing missing values, the nature, or mechanism, of the missingness is important. Missing data mechanisms can be categorized into three types: missing completely at random (MCAR), missing at random (MAR), or missing not at random (MNAR) [6,7]. Simple methods such as listwise deletion or mean imputation will only be unbiased when data are MCAR. Most current imputation methods assume MAR in order to produce unbiased results. Unfortunately, proving that the pattern of missingness in a real world dataset is MAR, without background knowledge of the actual mechanism itself, is impossible [8]. Indeed, most real world scenarios likely involve some degree of MNAR, where whether a value is missing or not depends on the value itself. In the best case scenario, this pattern of missingness can be modeled using prior knowledge in order to bring the data closer to MAR and improve the quality of imputations obtained through methods that assume MAR.

We briefly summarize several popular groups of machine learning methods for imputation. Of the four methods discussed below, nearest neighbors and self-organizing maps are clustering algorithms while decision trees and graphical models are classification algorithms. For more details on the design and implementation of these methods for imputation, we refer the reader to a review by Garcia-Laencina et al. (2009) [9].

### 2.2. Nearest Neighbors

In order to impute a value of a variable for a given sample, k-nearest neighbors (k-NN) takes a weighted average of the variable from the k closest samples. In comparison to many traditional techniques, k-NN has the advantage of being usable with mixed continuous and categorical data. k-NN is similar to hot deck imputation, a traditional imputation method, in the sense that similar examples are used to fill in missing values. Indeed, hot deck with a distance-based donor pool of size one is equivalent to 1-nearest neighbor. Hot deck typically picks randomly amongst similar examples to determine which value to fill in, whereas k-nearest neighbor typically uses a weighted mean (continuous) or a mode (categorical) to determine values.

### 2.3. Self-Organizing Maps

Self-organizing maps (SOMs), or Kohonen maps, are a class of neural network where neighboring nodes compete with each other to learn specific patterns in an unsupervised fashion [10]. SOMs have the ability to ignore variables with missing values during training. SOM imputation takes advantage of this fact by first training a map, then passing a sample with missing data to the map, observing the group that gets activated, and using the weights of the nodes in the activation group to determine the values to impute for the missing variables [11]. Because self-organizing maps handle missing data during training, it, like k-NN, possesses the advantage of only needing to train one model regardless of how many variables have missing data.

### 2.4. Decision Trees

Decision trees make supervised classifications from categorical or discretized data. A popular implementation is Quinlan’s C4.5 [12] algorithm, which inherently handles missing values by ignoring them when calculating information gain. Thus, as an imputation method, C4.5 can be trained when missing data are present in the predictor variables, increasing the potential training set available in the multivariate missing value setting.

### 2.5. Bayesian Networks

Bayesian networks capture probabilistic relationships between variables in a concise manner by enforcing conditional independence constraints. They can be constructed through a heuristic search using a Bayesian scoring function such as K2 [13]. Using Bayesian networks for imputation has several advantages: One, it is more efficient than MCMC or EM-based multiple imputation methods for a dataset with a large number of variables; two, it preserves the joint probability distribution of the variables, something that methods like k-NN do not promise. Unfortunately, a large amount of data are usually required to accurately learn a network, and discretization of all data is usually required unless conditional probability densities are explicitly modeled and parameterized, often at great computational expense [14,15].

### 2.6. Past Performance of Machine Learning Imputation Methods

We reviewed 12 papers that compared the performance of different imputation methods; they are summarized in Supplementary Table S1, with information on methods and their evaluation, along with types of datasets used and performance results reported. From our review, we observe that no imputation method consistently outperforms every other. Indeed, in several studies, the choice of imputation method seemed to have little impact on performance. We conclude that the nature of the dataset may have a larger impact on the performance of imputation methods than the imputation method itself.

### 2.7. Dealing with Missing Not at Random

Finally, we would like to briefly touch upon the case of data missing not at random. Without outside knowledge, it is impossible to determine if the mechanism is MNAR from data alone. In practice, however, researchers can make an educated guess based on expert knowledge of their domain as to whether a MNAR mechanism exists or not. In real world data, MNAR can occasionally be determined when data are “missing but known”, such as questionnaire data that was obtained after multiple reminders [16]. In this case, data obtained after a reminder were treated as missing for the purposes of analysis, though they were available at a later point. In a clinical trial setting, Kang et al. (2015) have proposed a Masked Missing Not at Random (MMNAR) assumption, where whether a clinical outcome is missing or not might depend on the outcome itself, but not on the assigned treatment due to clinical trial masking (also known as blinding) [17]. The authors showed that maximum likelihood imputation assuming MMNAR had smaller biases than the same imputation assuming MAR in a simulated MMNAR dataset. Although this method does not address the MNAR problem generally, it demonstrates that we can reduce the impact of MNAR models through limited assumptions. Similarly, Little, Rubin, and Zangeneh have recently introduced the idea of partial MAR and conditions for ignoring the missing data mechanism [18].

## 3. Materials and Methods

This study was approved by the University of Pittsburgh Institutional Review Board (IRB). Echocardiography and cardiac MRI text reports from pediatric patients (ages from 3 days to 22 years) undergoing evaluation for cardiomyopathy or myocarditis were obtained from the University of Pittsburgh Medical Archival System (MARS). A text report was obtained for each patient from their initial cardiac MRI work up for cardiomyopathy or myocarditis. Only the most recent echocardiography report prior to that cardiac MRI was taken. Quantitative cardiac measurements were extracted from the echocardiography reports and used as feature variables (Table 1). Cardiac MRIs reports were read to determine if they were positive or non-positive for cardiomyopathy, which was used as our target variable for classification. For more details on these methods, please refer to our previous paper [3].

In our previous work, we used listwise deletion on the full dataset (*n* = 88) to obtain a complete dataset (*n* = 50). In this paper, we use imputation methods to obtain estimates for the remaining data (*n* = 38), which we will call the imputed dataset. Each sample in the imputed dataset would have had at least one variable for which its value was missing. The percentage of missing data for each variable in the positive and non-positive MRI groups is shown in Table 1. Significant differences in the proportion of missing values for a given variable may make it easier to impute one group versus the other.

We sought to determine whether incorporating imputed data when training a Bayesian Rule Learning (BRL) classifier can increase its performance for this cardiomyopathy prediction problem. Because we have no ground truth available for our dataset, it is impossible to determine the bias of the imputed data themselves. Instead, we will measure the success of imputation methods solely by the sensitivity and specificity of models learned using the imputed data.

For clarity, we will refer to the different sets of data as full data or imputation-augmented data (*n* = 88), complete data (*n* = 50), and imputed data (*n* = 38). We refer to models learned on the full data as imputation-augmented models and models learned on the complete data as unaugmented models. In the context of BRL, the term model refers to a ruleset. We imputed missing values using four methods: Mean imputation, k-nearest neighbors, C4.5 decision trees, and self-organizing maps. Out of the four methods, decision tree is a classification-based method, k-nearest neighbors and self-organizing maps are clustering-based, and mean imputation was chosen as a simple baseline to compare against. We did not use Bayesian networks for imputation as our dataset is too small to learn an accurate network. Of the four methods used, all are deterministic except for self-organizing maps, which starts with a set of randomly initialized weights but converges to an optimum weight vector. As such, multiple imputation is not feasible with the imputation methods we are exploring. In addition, because no ground truth was available, we compared imputed values across imputation methods to identify any differences between the methods.

In our previous work, variable pruning was necessary to remove variables that had too many missing values. This resulted in a hand-picked set of 14 variables that still captured both cardiac function and morphology. Before variable pruning, we had a set of 36 which included both variables and their calculated indices or z-scores. The full set of 36 variables were used for initial imputation.

Before imputation, all variables were continuous. In the case of decision tree imputation, all variables had to be discretized before a decision tree could be trained. In this case, values were discretized in one of two ways: Variables that were calculated z-scores were discretized into Low, Normal, or High based on a z-score of less than −3, between −3 and 3, and greater than 3, respectively. All other variables were discretized into 3 weighted frequency bins, with weights of 25%/50%/25%. Decision tree imputation was implemented in Java using Weka 3.9 [19].

K-nearest neighbors were implemented in Matlab. All variables were normalized to mean 0 and standard deviation 1. A Euclidean metric was used to calculate distance between samples, and any variables that contained a missing value in either sample were ignored for the calculation. The contributions of the top k neighbors to the imputed value were weighted based on distance.

SOM imputation was performed using the Imputation SOM algorithm described in Vatanen et al. (2015) and implemented in the SOM Toolbox for Matlab [20]. Mean imputation was implemented in Matlab.

**Figure 2:**
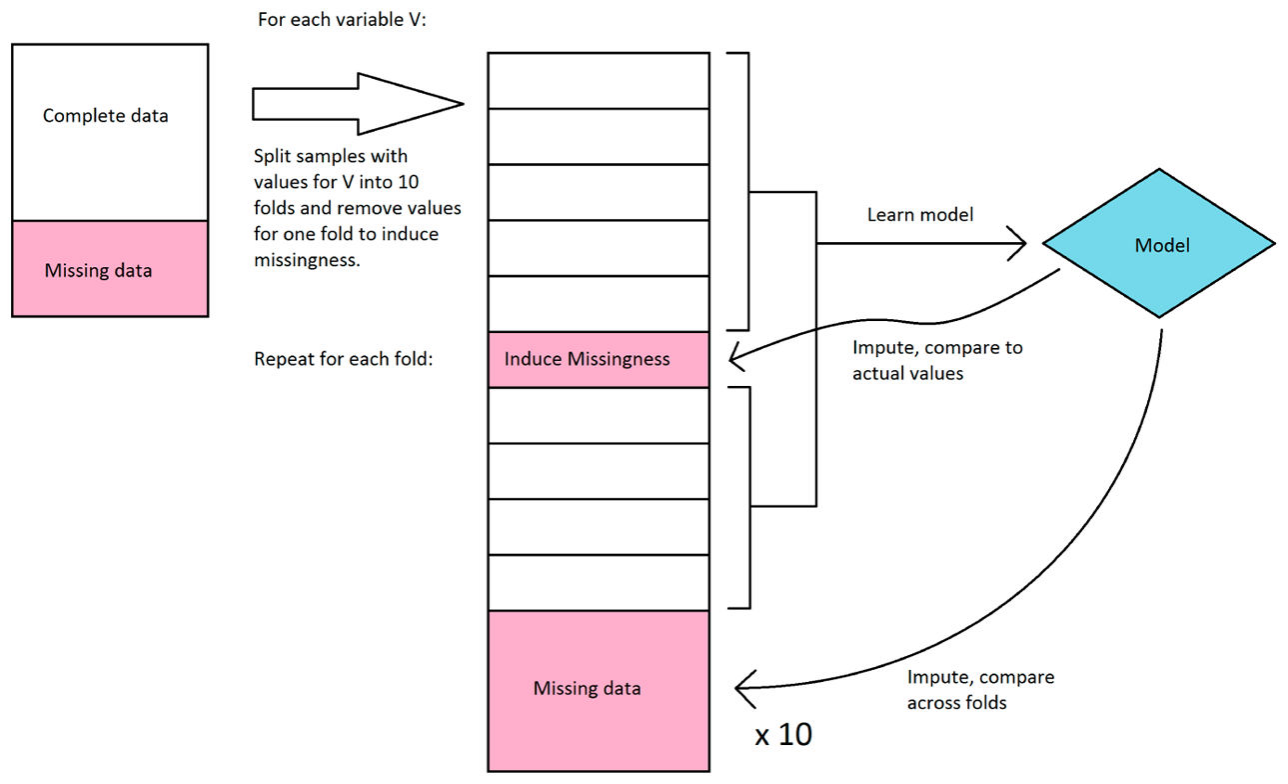
Workflow diagram of evaluation of decision tree imputation.

We first explored the stability of our only classifier-based imputation method, decision tree imputation. Because we have no ground truth to evaluate the quality of the imputation against, we instead sought to see how much the imputed values changed if we learned the trees on a subset of the available data. Decision tree imputation requires learning a model for each variable to be imputed. For each variable, we split the samples that had values for that variable into 10 sets, and learned a decision tree model for every combination of 9 sets (in effect, 10-fold cross-validation). These models were then used to impute values for the remaining set as well as the samples that were missing values. We compared the imputed values for each set to the actual values, and we compared imputed values for the samples with missing values across the models learned in each fold. Because decision tree imputation works on discrete bins, we calculated accuracy and agreement instead of error and variance. For the samples that had values, we compared the accuracy of the imputed values compared to the actual values. We disregarded bin order in this calculation, so a prediction was considered incorrect if it was in a different bin, regardless of how far the bin was from the actual bin. For the samples that were missing values, we compared the agreement of imputed values across the 10 folds. The agreement was calculated as follows: For each sample, the percent agreement is the percent of the majority bin imputed across the 10 folds. The overall agreement is the mean of the percent agreement across all samples with missing values. We did not use Fleiss’ kappa because it misrepresents agreement when most or all samples were imputed in the same bin. This workflow is shown in Figure 2.

We then used BRL to learn models for each imputation-augmented dataset and compared their performance to models learned on just the complete data. The best performing configuration for BRL in our previous work was used for all experiments. This included using efficient Bayesian discretization [21] with a lambda of 10. For the unaugmented model, performance was evaluated on sensitivity and specificity in 10-fold cross validation. Five separate randomized runs of cross validation were done to obtain an average performance.

**Figure 3:**
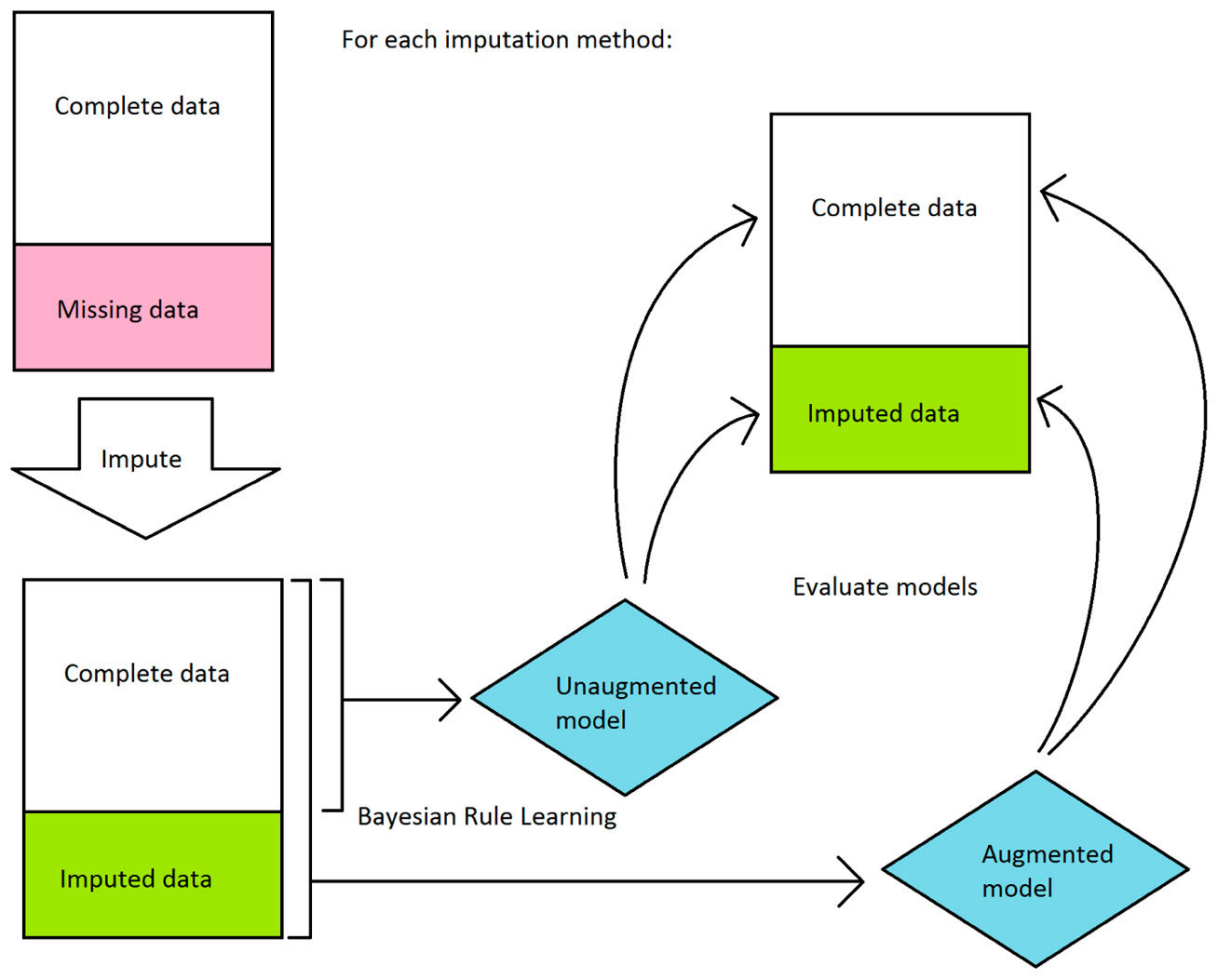
Workflow diagram of evaluation of imputation-augmented models.

In order to compare the performance of imputation-augmented models to the performance of our previous unaugmented model, we needed to evaluate using the same dataset. Since the unaugmented model was evaluated on just the complete data, we needed to split the evaluation of our imputation-augmented models between performance on complete data versus performance on imputed data as well. An overview of the workflow is shown in Figure 3. We thus aggregated predictions for each sample from 10-fold cross validation and then split them into complete data versus imputed data predictions. Sensitivities and specificities were then calculated separately for each group. This process was repeated 5 times for 5 different randomized 10-fold cross-validations, and the sensitivities and specificities were averaged over the 5 runs. The same 5 randomized cross-validation splits were used for each imputation method. Because the BRL ruleset classifier does not normally produce a score that can be thresholded to produce an area under the receiver operating characteristic curve(AUC), we calculated AUCs using the posterior probabilities *pr_post_* for each rule fired by a test sample. For samples that triggered negative rules, 1 − *pr_post_* was used instead, so that the probabilities could be interpreted as the probability of predicting positive. This probability was then thresholded to produce an AUC using trapezoidal approximation. Like sensitivity and specificity, the AUC was averaged over 5 cross-validation runs.

In order to estimate the performance of an unaugmented model on imputed data, we took the parsimonious ruleset learned from the whole training set of complete data and applied it to the imputed data. This could only be done to the k-NN, SOM, and mean imputation augmented datasets, as decision tree imputation required discretization before imputation and thus had different discretization ranges compared to the unaugmented model.

To test the hypothesis of whether increasing sample size improves performance, we learned imputation-augmented models using only the 14 variables used previously in learning our unaugmented models. To test the hypothesis of whether increasing sample size and variable count improves performance, we learned imputation-augmented models using data that contained extra variables that were previously thrown away. This dataset consisted of the full 36 variables, minus variables that were also present in the dataset in the form of an index or z-score (e.g., End Diastolic Volume removed in favor of End Diastolic Volume Index, Interventricular Septum Thickness removed in favor of Interventricular Septum Thickness z-score), resulting in 27 variables total. These variables are defined in Table 1.

## 4. Results

The proportion of positive to non-positive samples was 0.36 in the complete data set (*n* = 50) and 0.35 in the full data set (*n* = 88).

**Figure 4:**
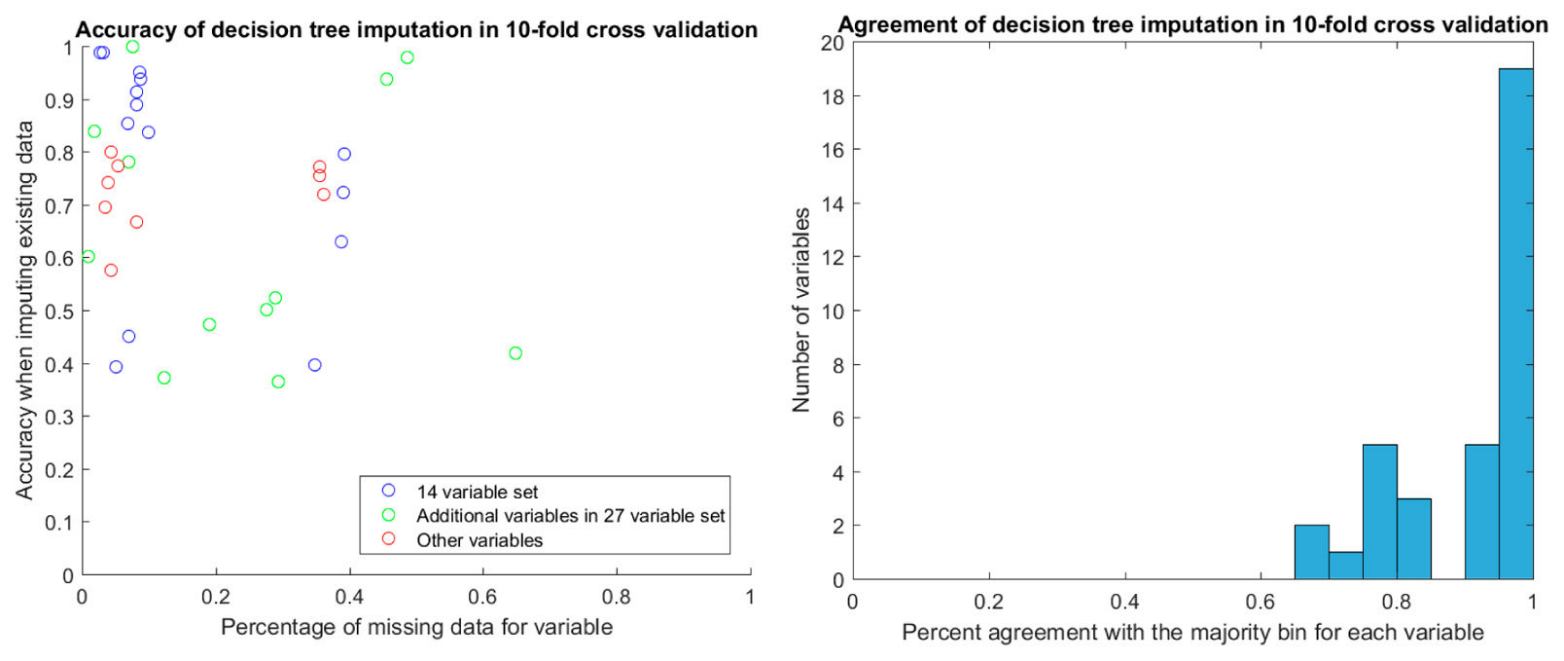
Accuracy and agreement of decision tree imputed values in 10-fold cross validation. Accuracy was calculated from the imputed values for the samples that had values, while agreement was calculated from the imputed values for the samples that did not have values.

After discretization, we evaluated the stability of decision tree imputation using 10-fold cross-validation. The accuracy of imputed values in cross-validation ranged between 0.4 and 1, depending on the variable. There was no relationship between the percentage of missing data for the variable and the accuracy of the imputation (Figure 4). Of particular note are the two additional variables in the 27-variable set (green circles in Figure 4) that have around 50% missing values yet high imputation accuracy in cross-validation. These are TR max PG and TR max velocity, which feature prominently in the decision-tree augmented rulesets that we will discuss later. With the exception of two samples, TR max PG and TR max velocity were either both present or both miss for a given sample.

For the values that were missing, we found the agreement of imputed values to the majority value was high, with the vast majority agreeing perfectly. The majority of these imputed values were in the discretized bin that covered the mean for the variable.

**Figure 5:**
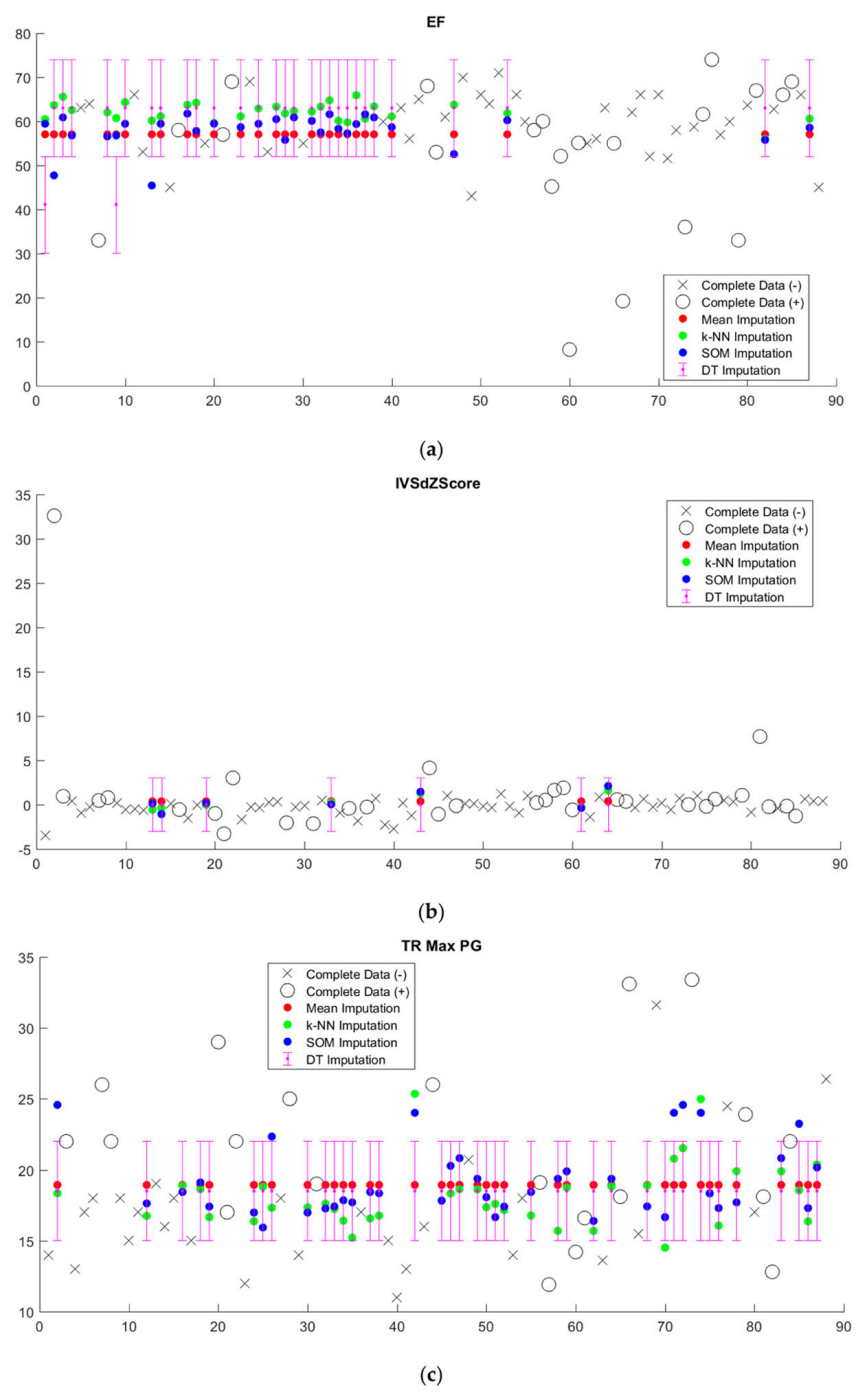

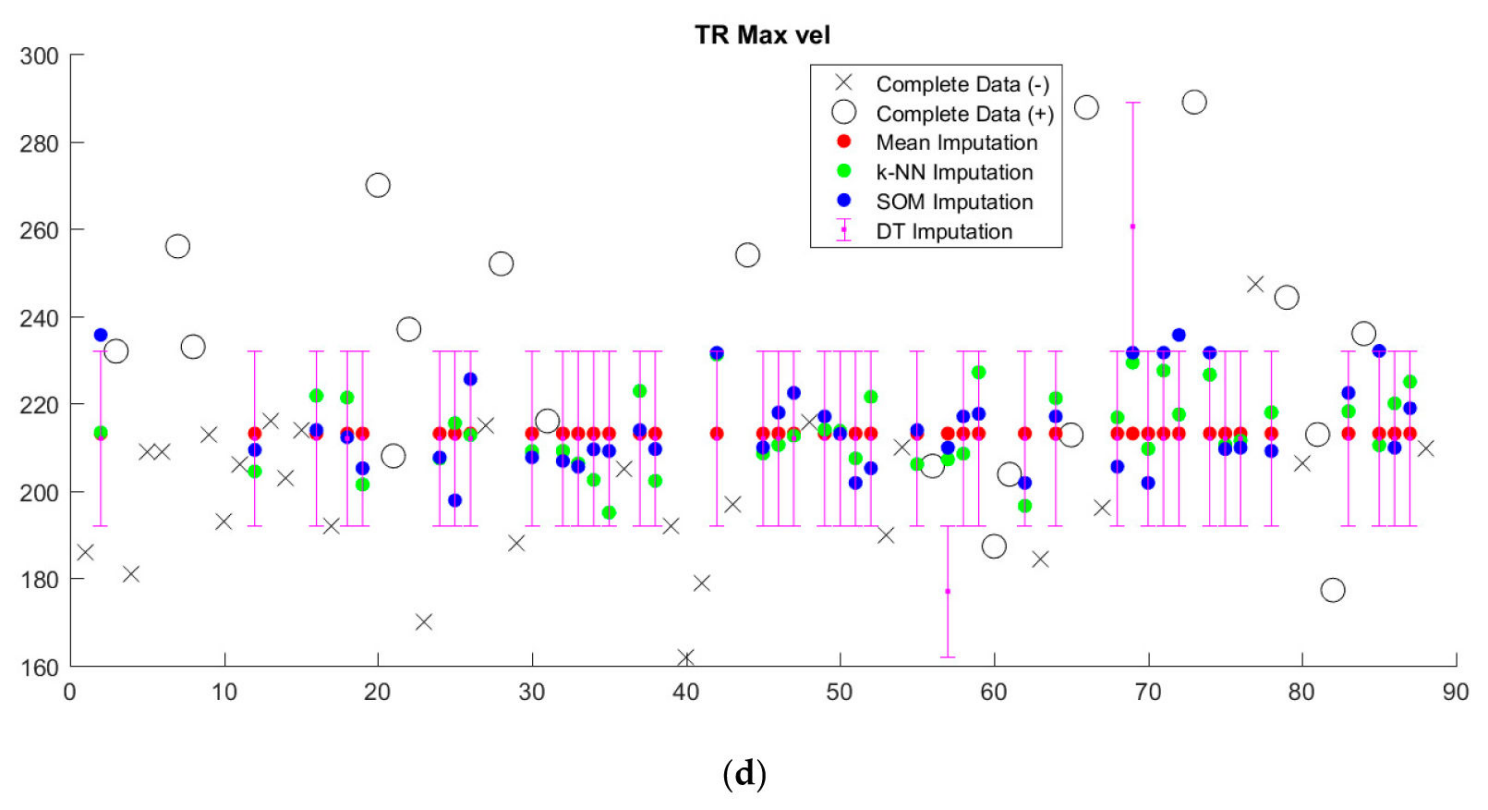
Imputed values for four representative variables (**a**) ejection fraction (EF), (**b**) interventricular septum thickness z-score (IVSdZScore), (**c**) tricuspid regurgitation max pressure gradient (TR Max PG), and (**d**) tricuspid regurgitation max velocity (TR Max vel). Observed values for the positive class are shown as black circles and observed values for the negative class are shown as black X’s. Imputed values for mean, k-NN, and SOM imputation are shown as red, green, and blue dots, respectively. Because decision tree (DT) imputation requires discretized values, imputed values are reported as a discretized range.

Imputed data tended to cluster near the mean, regardless of imputation method (Figure 5, Supplementary Figure S1). Since we sought to limit the number of discretized bins, discretization washes out small variations in imputed values. This is particularly apparent in the case of decision tree (DT) imputation, which requires pre-discretization. Because BRL requires discretized input data, the small variations in imputed values from k-NN and SOM imputation from the mean will in most cases be washed out as well.

**Figure 6:**
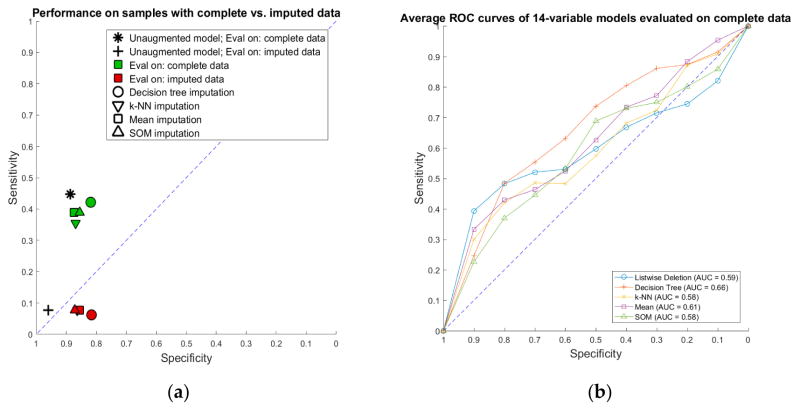
Performance of imputation-augmented rulesets compared to unaugmented rulesets: (**a**) sensitivity vs. specificity of 14-variable models evaluated on complete vs. imputed data; and (**b**) average receiver operating characteristic (ROC) curves of 14-variable models evaluated on complete data.

Performance of imputation-augmented models with 14 variables evaluated on complete data was similar to that of an unaugmented model on complete data (Table 2, Figure 6). We found no significant difference, after Bonferroni correction for multiple comparisons, between the rulesets learned after imputation versus the rulesets learned from complete data. Taking the whole training set rules learned in our previous paper [3] and applying them to the imputed data, we achieve a specificity of 0.96 and a sensitivity of 0.08 on data imputed through the k-NN, mean, and SOM imputation methods. Each of the three imputation methods produced values that resulted in identical classifications with the old ruleset, so they are displayed as one point in Figure 6. Performances of all models evaluated on imputed data were all poor.

**Figure 7:**
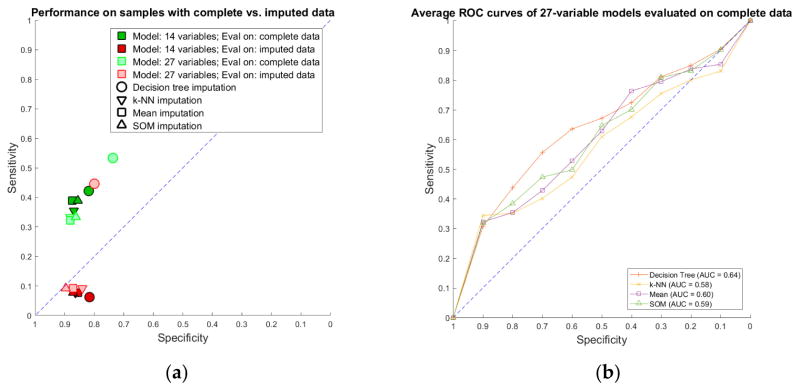
Performance of 27-variable rulesets compared to 14-variable rulesets: (**a**) sensitivity vs. specificity of 27-variable models compared to 14-variable models evaluated on complete data vs. imputed data; and (**b**) average ROC curves of 27-variable models evaluated on complete data.

Imputation-augmented models learned using 27 variables performed similarly to models learned using 14 variables when evaluated on complete data (Figure 7). With the exception of decision tree imputation, all models performed similarly poorly on imputed data. Decision tree imputation-augmented models learned using 27 variables performed just as well on imputed data as on complete data. This was due to the inclusion of TR max velocity or TR max PG in the additional variables. An average of 97% of samples predicted using cross validation from the decision tree 27-variable imputed data used TR max velocity or TR max PG in their rules, compared to an average of 10% for the other imputation methods. Upon further examination, we found that this was not because decision tree imputation did a better job with imputing these variables (the imputed values for TR max PG and TR max vel are shown in Figure 5). Rather, the increased performance was due to the different discretization thresholds calculated as a result of discretizing pre-imputation for decision trees versus post-imputation for everything else. When discretization thresholds were calculated pre-imputation, the other 27-variable imputation-augmented models performed similar to decision tree on the imputed data. Doing the same for the 14-variable imputation-augmented models produced no significant difference in performance, however (Supplementary Figure S2).

Finally, we compared the number of rules learned on the 14-variable and 27-variable whole training sets for each method (Table 3). There is a polynomial relationship between the number of discrete bins per variable and the number of rules, and thus the choice of discretization method directly affects the number of rules. The unaugmented ruleset contained seven rules using two variables: ejection fraction (EF) and interventricular septum thickness (IVSd z-score). The rules learned on the mean, k-NN, and SOM imputed datasets with 14 variables were the same, and contained 15 rules using four variables, interventricular septum thickness, left ventricular internal dimension, left ventricular mass, and body surface area. Decision tree imputation, unlike the other methods, used unsupervised discretization pre-imputation, resulting in on average more bins per variable than the efficient Bayesian discretization method used for the other methods. The rules learned on the decision tree imputed dataset produced 183 rules using nine variables. The presence of IVS thickness in all the rulesets suggests it is of particular importance. Only 8% of the values for this variable were missing. On the other hand, 34% of EF values were missing, which make its absence from mean, k-NN, and SOM imputed rulesets not surprising.

Rulesets learned on the 27-variable whole training set, unsurprisingly, contained more rules compared to those learned on the 14-variable whole training set. The presence of IVSd z-score in all these rulesets confirms its importance. What is notable, however, is the addition of tricuspid valve related measures (TV A max, TV E max, TV E/A, or TR max vel) from the additional variables. Their presence is surprising, as the majority of our positive cardiomyopathy cases did not primarily affect the right side of the heart.

## 5. Discussion

We found similar performance of imputation-augmented models to unaugmented models when evaluated on both complete and imputed data. Predicting imputed data was much harder compared to predicting complete data in our dataset. In particular, sensitivity suffered the most when it came to classifying the imputed samples. We erroneously predict a non-positive MRI for cardiomyopathy more frequently in the imputed sample set compared to the complete sample set. There did not appear to be a strong enough relationship between variables to push clustering-based estimates (k-NN, SOM) away from the mean. For the most part, decision tree imputation failed to predict values far from the mean as well. Because every imputation method resulted in values that fell close to the mean (or was the mean in the case of mean imputation), BRL rules that predict positive MRIs based on abnormal values would tend to classify samples with imputed data as normal, resulting in low sensitivity.

The choice of imputation method does not seem to drastically affect our results. This finding is in line with our conclusion from a review of the literature that no imputation method consistently outperforms any other. Indeed, in several cases including ours, mean imputation can achieve results comparable to much more complicated algorithms. In our case, discretization eliminates much of the variance between imputation methods, washing out small differences in imputed values. As such, simple imputation methods performed just as well as more sophisticated ones.

The choice of discretization method in our case seems to change which variables BRL learns. Using the expanded 27-variable data set, unsupervised, weighted frequency binning resulted in BRL picking up TR max velocity and TR max PG as important variables, which significantly increased the performance of the learned rulesets on the new 38 samples. The inclusion of these variables is made possible with imputation. Unfortunately, the inclusion of these variables did not significantly improve performance on the original 50 samples. The increase in performance due to these two measurements is particularly puzzling, as the vast majority of our positive cardiomyopathy cases are limited to the left ventricle with little or no right-sided involvement. On their own, TR max velocity and TR max PG can achieve AUCs close to those achieved by our multi-variate models. This is evidence that the importance of these variables is characteristic of our dataset, rather than an issue with over-fitting of our models. Unfortunately, because our dataset is so small, this could very well be a bias in our dataset with no generalizability. We will need more data to investigate whether the presence of right-sided heart measurements in left-sided cardiomyopathies is clinical or merely an artifact of the dataset.

Although the lack of increase in performance may make imputation seem like a waste of time, the consistency in performance of imputation-augmented models provides evidence for the robustness of our model learning mechanism. By analyzing the variability of rules and variables used, we can obtain a measure of which variables possess a strong association with our target variable and which do not. In our classification problem, we found that interventricular septum thickness was consistently associated with whether a subsequent cardiac MRI was positive or not for cardiomyopathy.

In the overall context of the clinical guideline learning problem, the small size of the data set is the major limitation. The small sample size both limits our statistical power and calls into question the applicability of our findings to the general population. Cardiac magnetic resonance imaging is a relatively new modality in the evaluation of pediatric cardiomyopathy and not part of the standard work-up, which limits the amount of data available for study. This lack of data motivated the exploration of imputation as a potential work-around.

Future work on imputation with this cardiac dataset must focus on expert knowledge-driven modeling of missing values. While recollecting data is impossible due to the retrospective nature of the dataset, imputation methods could be made more accurate by gathering additional variables likely to be related to the missing variables in question. This could take the form of the same variable measured at different time points, or clinically correlated variables with previously known relationships to the missing variable. An imputation method that relies on this becomes more akin to extrapolation or interpolation.

When answering the question of whether to impute or not, we offer several points of consideration. First, the percentage of missing values and their distribution across variables must be considered. Our echocardiography dataset had 15% missing values overall, but each variable ranged from 2% to 39% missing values. If an imputation method draws values from the sample variable in other samples (such as mean, k-NN, or SOM), the large number of missing values for a given variable is important. If an imputation method generates values from other variables altogether (e.g., decision tree imputation or other classification-based methods), then the number of missing values per variable might be less important. Second, even if imputed data do not improve model learning, it may still be useful for demonstrating the robustness of the model learning method to lower quality data. The inclusion of extra variables that previously would have been discarded might be a potential benefit as well. In light of these advantages, we believe that imputation provides useful information regardless of its impact on model performance. Taking into account the observation that simple imputation methods often perform just as well as more complex methods, we would recommend that imputation be performed for data with missing values in any situation where it might be feasible.

## 6. Conclusions

We review machine learning methods for missing value imputation and compare the performance of several imputation methods on a rule learning task. We found that simple imputation methods (mean imputation) and listwise deletion performed just as well as more complex imputation methods (decision trees, k-NN, and SOM). From our review and experiment, we conclude that the imputation provides useful information about the robustness of a model learning method regardless of its impact on model performance. Combined with the observation that simple imputation methods often perform as well as more complex ones, we would recommend imputing missing values whenever feasible.

## Supplementary Material

Supplemental1

Supplemental2

## Figures and Tables

**Table 1:** Variable definitions for the 14-variable and 27-variables and what percentage of each variable was missing in the positive (+) versus the non-positive (−) MRI group.

Variables in 14 Variable Set	Definition	Percentage Missing (+)	Percentage Missing (−)
BSA	Body Surface Area	3.2%	8.8%
EDV index	End diastolic volume index	38.7%	38.6%
ESV index	End systolic volume index	38.7%	38.6%
SV index	Stroke volume index	38.7%	38.6%
FS	Fractional shortening	3.2%	5.3%
EF	Ejection fraction	32.3%	35.1%
Ao V2 max	Aortic V2 max	3.2%	1.8%
Ao max PG	Aortic max pressure gradient	3.2%	1.8%
MV E/A	Mitral valve E/A ratio	16.1%	1.7%
IVSd z-score	Interventricular septum thickness measured in diastole, z-score	3.2%	10.5%
LVIDd z-score	Left ventricular internal dimension measured in diastole, z-score	3.2%	10.5%
LVIDs z-score	Left ventricular internal dimension measured in systole, z-score	3.2%	12.3%
LVPWd z-score	Left ventricular posterior wall thickness measured in diastole, z-score	3.2%	10.5%
LV mass z-score	Left ventricular mass measured in diastole, z-score	3.2%	10.5%

**Additional Variables in 27 Variable Set**	**Definition**	**Percentage Missing (+)**	**Percentage Missing (−)**

Age	Age at scan	0%	0%
Height	Height at scan	3.2%	8.8%
Weight	Weight at scan	0%	1.8%
Ao root diam	Aortic root diameter	35.5%	22.8%
MV A max	Mitral valve A wave max (max atrial filling velocity)	35.5%	24.6%
MV E max	Mitral valve E wave max (max early filling velocity)	38.7%	22.8%
PA V2 max	Pulmonary artery V2 max	12.9%	3.5%
PA max PG	Pulmonary artery max pressure gradient	12.9%	3.5%
TR max PG	Tricuspid regurgitation max pressure gradient	35.5%	50.9%
TR max vel	Tricuspid regurgitation max velocity	38.7%	52.6%
TV A max	Tricuspid valve A wave max (max atrial filling velocity)	25.8%	14.0%
TV E max	Tricuspid valve E wave max (max early filling velocity)	19.4%	7.0%
TV E/A	Tricuspid valve E/A ratio	64.5%	64.9%

**Table 2:** Sensitivity, specificity, accuracy, and AUC of BRL rules learned on 14 variables using imputation-augmented data versus unaugmented data evaluated on complete data only, averaged over five 10-fold cross-validations. Performance metrics are tested against the performance of the unaugmented model. After Bonferroni correction for multiple comparisons, *α* = 0.0125 is the significance threshold (significant values denoted by *).

Method	Sensitivity	Specificity	Accuracy	AUC
Unaugmented model	44.7 +/− 4.7	88.7 +/− 5.1	73.5 +/− 3.4	59.2 +/− 6.5
Mean imputation	38.9 +/− 6.1 (*p* = 0.17)	87.5 +/− 4.0 (*p* = 0.71)	70.0 +/− 1.8 (*p* = 0.11)	60.8 +/− 2.9 (*p* = 0.66)
Decision tree imputation	42.2 +/− 7.5 (*p* = 0.61)	81.9 +/− 5.4 (*p* = 0.10)	67.6 +/− 4.5 (*p* = 0.07)	65.8 +/− 4.8 (*p* = 0.14)
k-NN imputation	35.6 +/− 5.7 (*p* = 0.04)	86.9 +/− 5.0 (*p* = 0.61)	68.4 +/− 3.25 (*p* = 0.07)	57.6 +/− 1.2 (*p* = 0.66)
SOM imputation	38.9 +/− 3.5 (*p* = 0.08)	85.6 +/− 4.7 (*p* = 0.39)	68.8 +/− 3.3 (*p* = 0.08)	57.8 +/− 1.9 (*p* = 0.70)

**Table 3:** Rules learned on the whole training set using 14 variables.

Method	Number of Rules Learned	Variables Used
Unaugmented model (14 variables)	7	EF, IVSd z-score (2)
Mean imputation (14 variables)	15	IVSd z-score, LVIDd z-score, LV mass z-score, BSA (4)
Decision tree imputation (14 variables)	183	IVSd z-score, LVIDd z-score, LVIDs z-score, LV mass z-score, EF, EDV index, SV index, MV E/A, Ao max PG (9)
k-NN imputation (14 variables)	15	IVSd z-score, LVIDd z-score, LV mass z-score, BSA (4)
SOM imputation (14 variables)	15	IVSd z-score, LVIDd z-score, LV mass z-score, BSA (4)
Mean imputation (27 variables)	43	IVSd z-score, LVPWd z-score, LVIDs z-score, MV A max, LV mass z-score, SV index, FS, TV A max, TV E max, height (10)
Decision tree imputation (27 variables)	255	Ao V2 max, EF, EDV index, FS, MV A max, PA V2 max, TR max vel, TV E/A, SV index, IVSd z-score, height, weight (12)
k-NN imputation (27 variables)	35	IVSd z-score, LV mass z-score, SV index, LVIDs z-score, MV A max, TV A max, TV E max, height (8)
SOM imputation (27 variables)	27	IVSd z-score, LV mass z-score, SV index, Ao root diam, LVIDs z-score, TV A max, TV E max, height (8)
