# Supplementary material for: An Overview and Evaluation of Recent Machine Learning Imputation Methods Using Cardiac Imaging Data
Source: Data (Basel). Author manuscript; Available in PMC 2017 Mar 1. (PMC5325161; doi:10.3390/data2010008)
Supplement: Supplemental1 [file NIHMS846826-supplement-Supplemental1.pdf]

Supplementary Materials: An Overview and Evaluation of Recent Machine Learning Imputation Methods Using Cardiac Imaging Data

Yuzhe Liu and Vanathi Gopalakrishnan

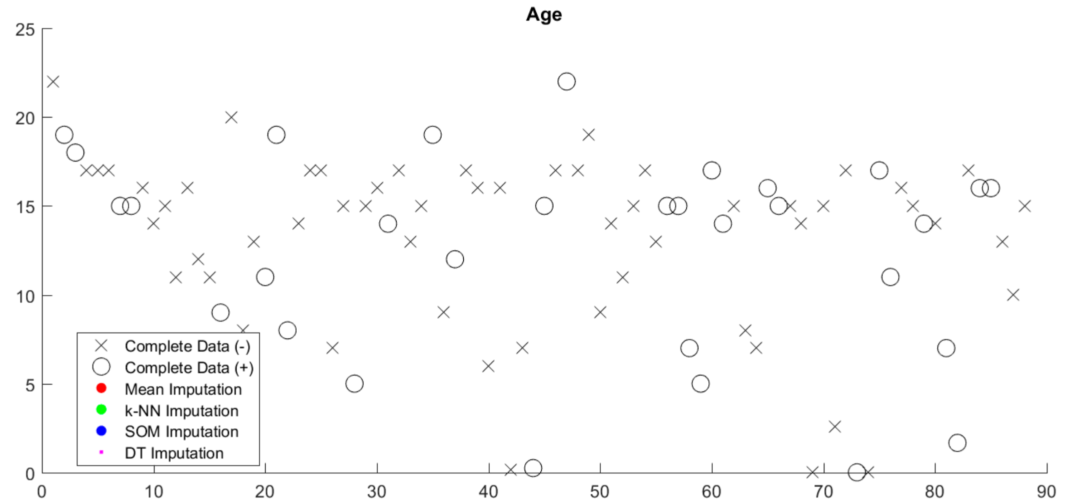

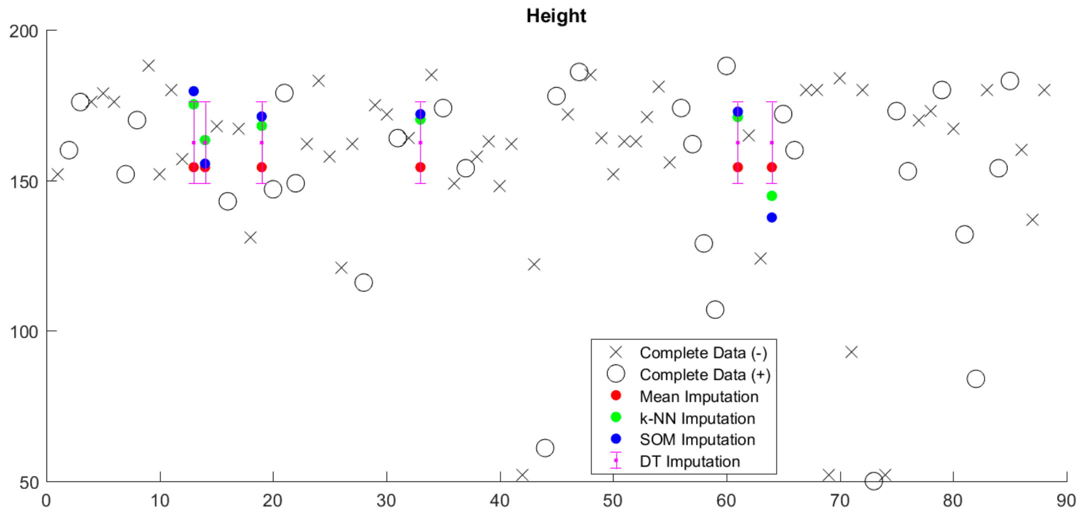

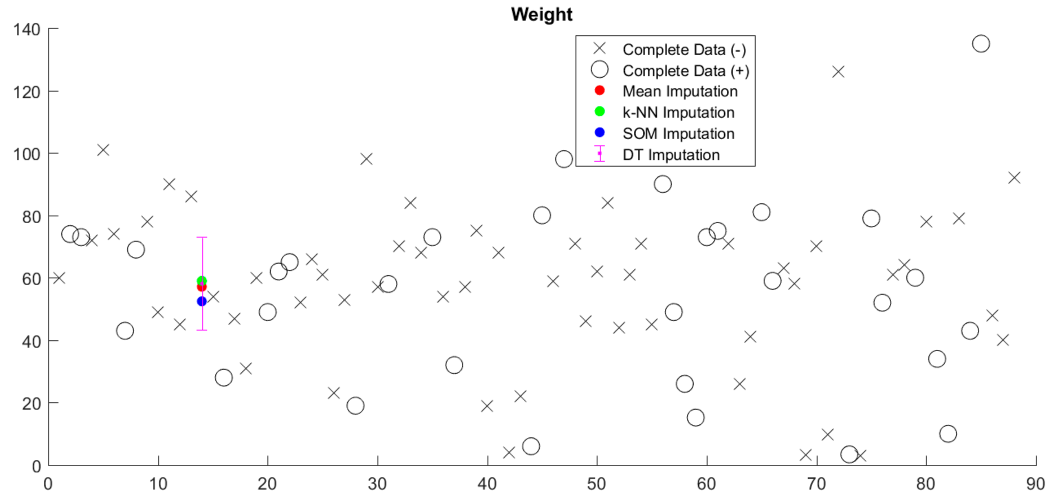

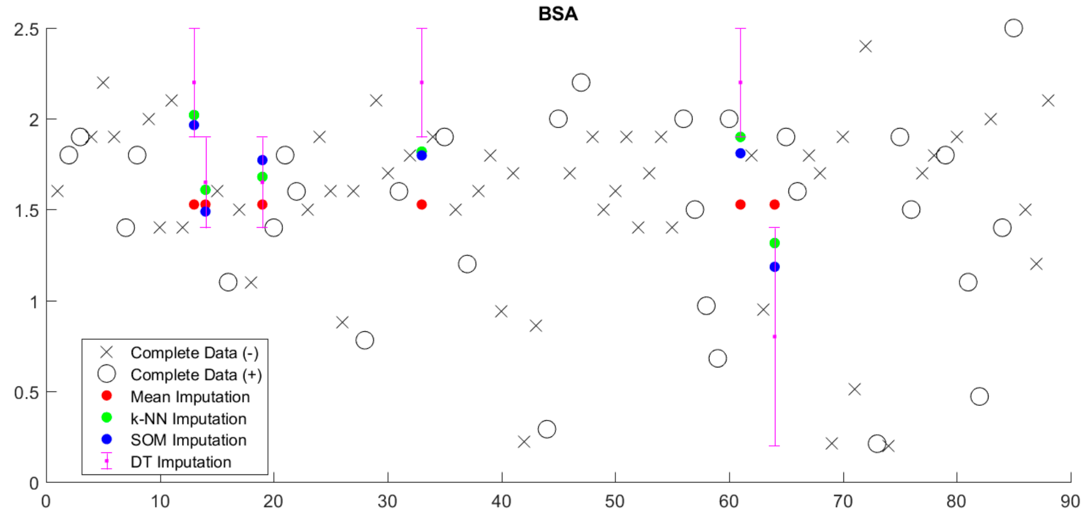
BSA
Complete Data (-)
Complete Data (+)
Mean Imputation
k-NN Imputation
SOM Imputation
DT Imputation

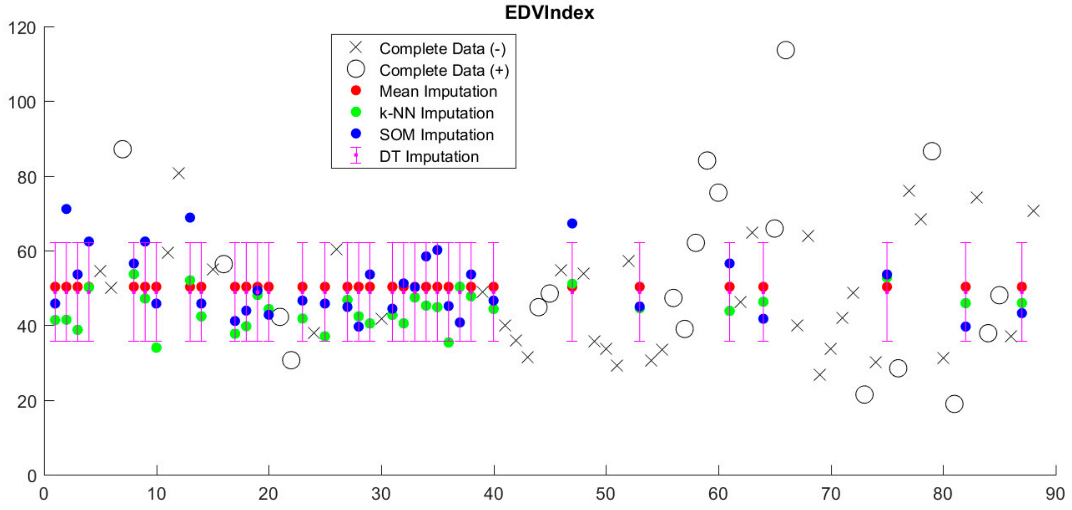
EDVIndex
Complete Data (-)
Complete Data (+)
Mean Imputation
k-NN Imputation
SOM Imputation
DT Imputation

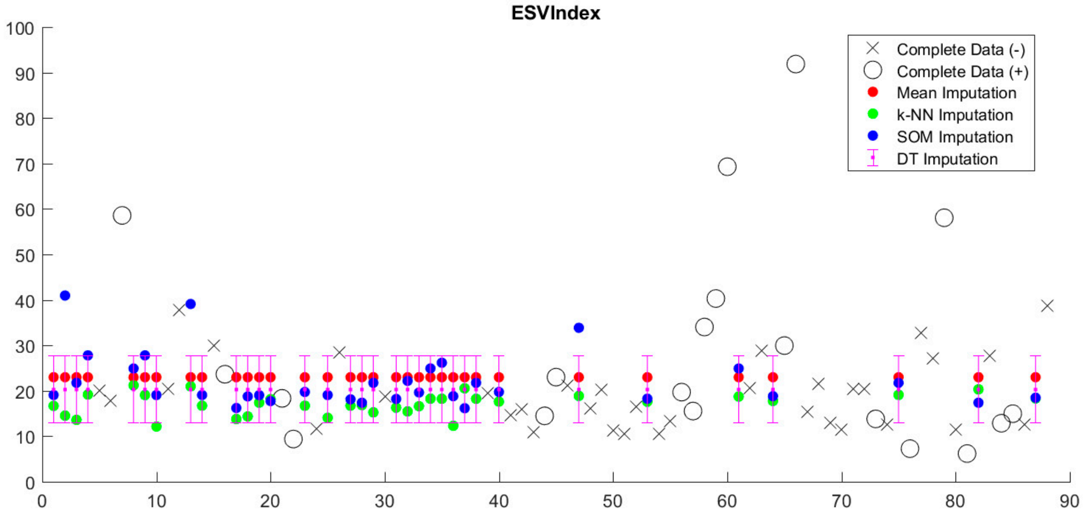
ESVIndex
Complete Data (-)
Complete Data (+)
Mean Imputation
k-NN Imputation
SOM Imputation
DT Imputation

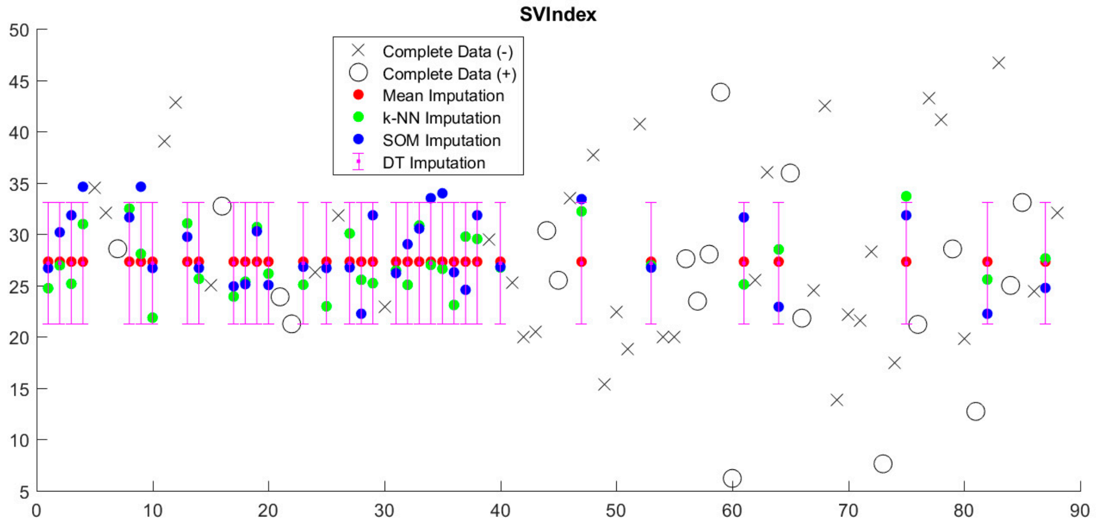
SVIndex
Complete Data (-)
Complete Data (+)
Mean Imputation
k-NN Imputation
SOM Imputation
DT Imputation

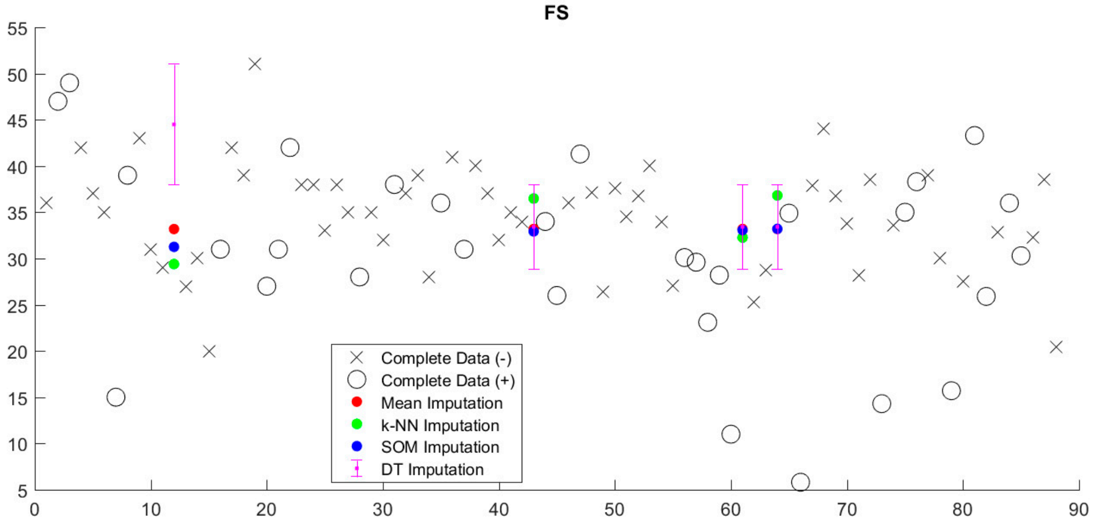
FS
Complete Data (-)
Complete Data (+)
Mean Imputation
k-NN Imputation
SOM Imputation
DT Imputation

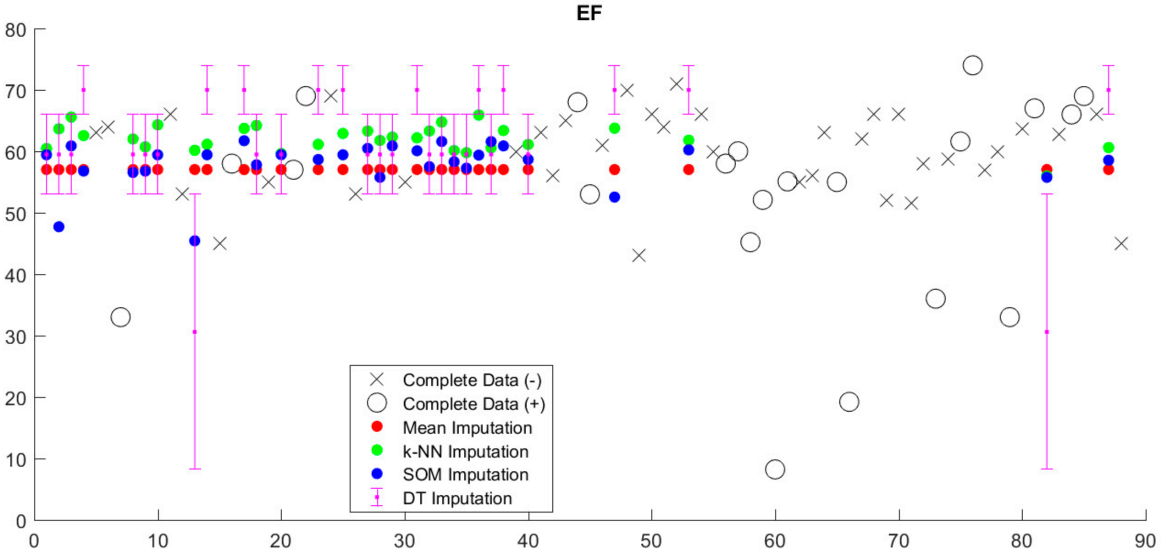
EF
Complete Data (-)
Complete Data (+)
Mean Imputation
k-NN Imputation
SOM Imputation
DT Imputation

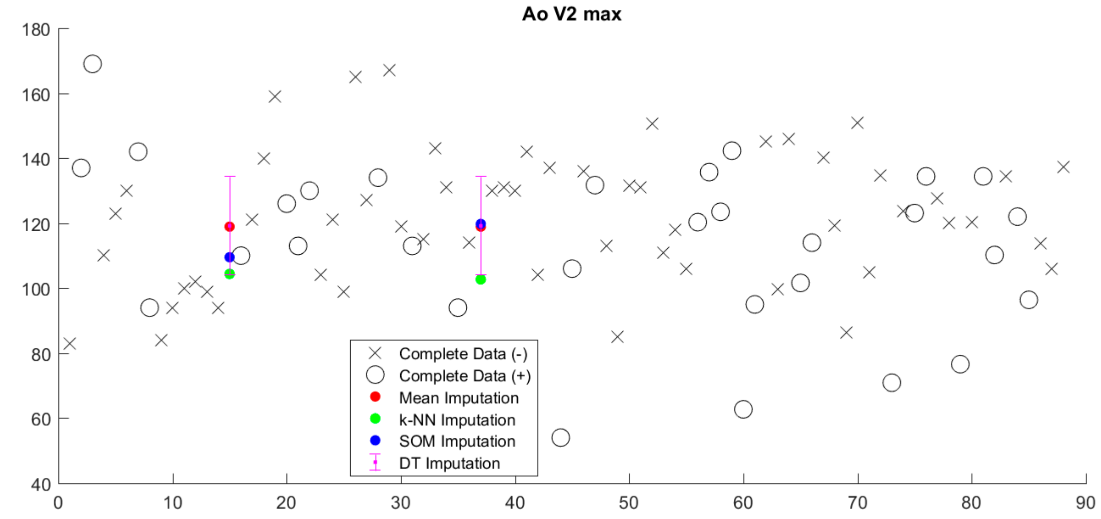
Ao V2 max
Complete Data (-)
Complete Data (+)
Mean Imputation
k-NN Imputation
SOM Imputation
DT Imputation

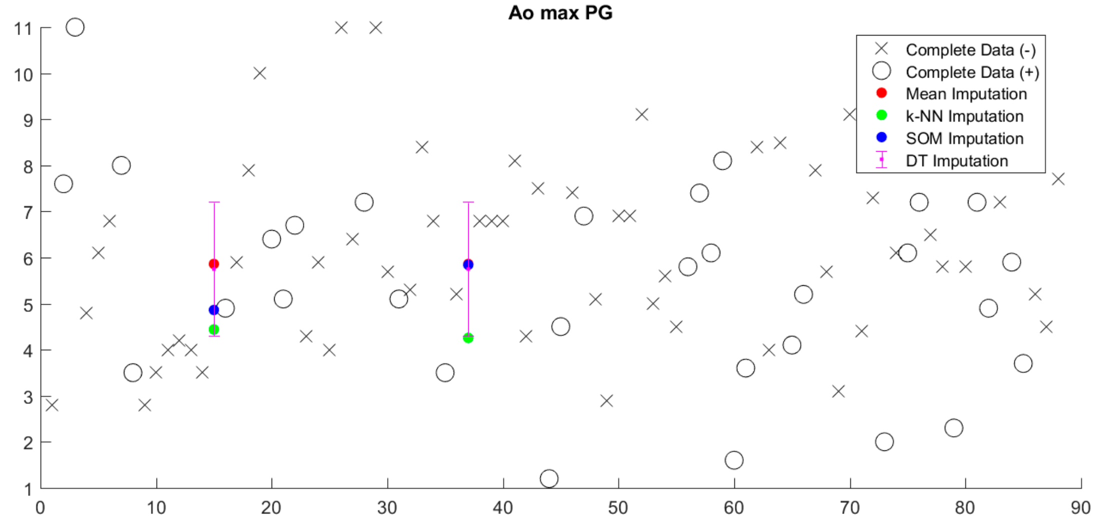
Ao max PG
Complete Data (-)
Complete Data (+)
Mean Imputation
k-NN Imputation
SOM Imputation
DT Imputation

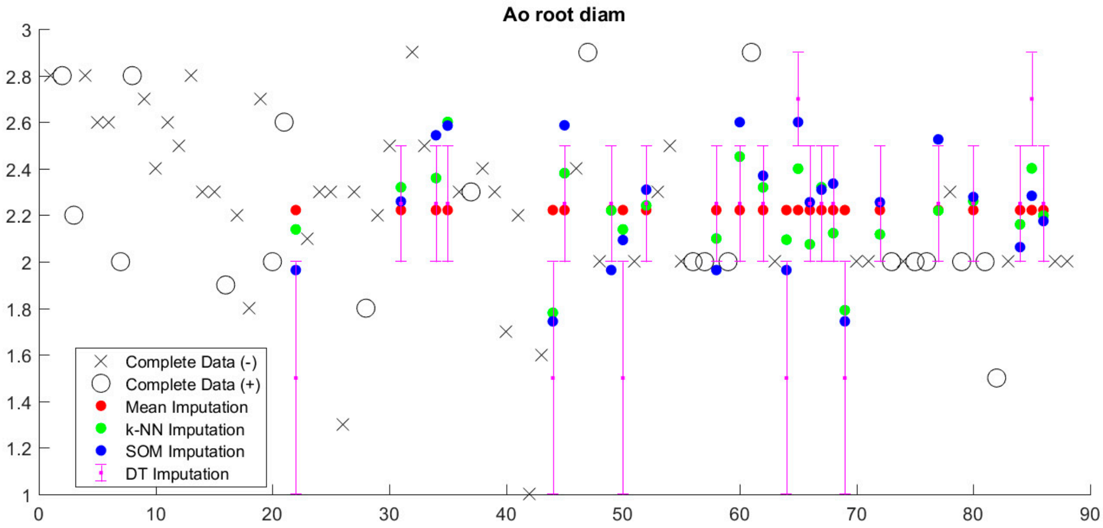
Ao root diam
Complete Data (-)
Complete Data (+)
Mean Imputation
k-NN Imputation
SOM Imputation
DT Imputation

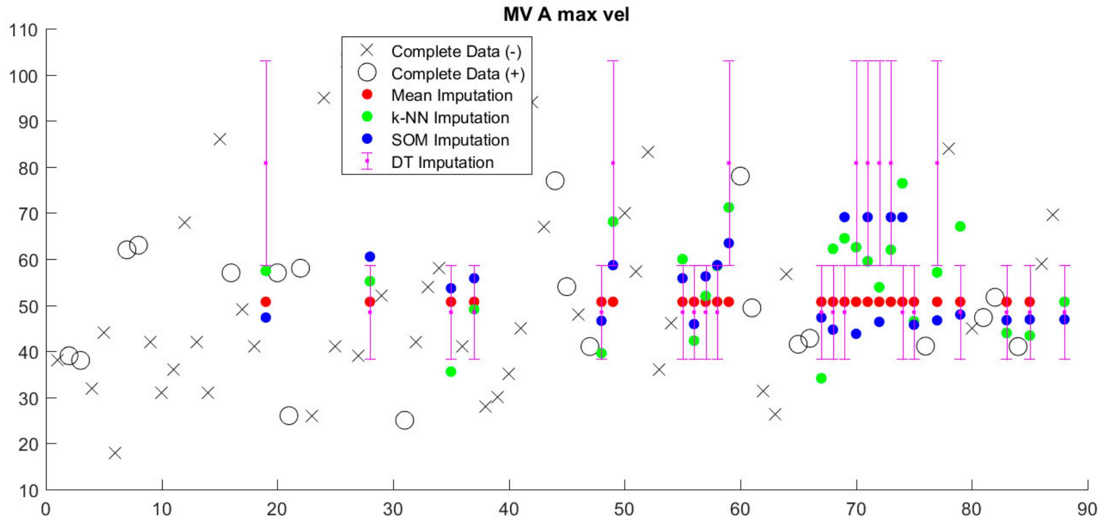
MV A max vel
Complete Data (-)
Complete Data (+)
Mean Imputation
k-NN Imputation
SOM Imputation
DT Imputation

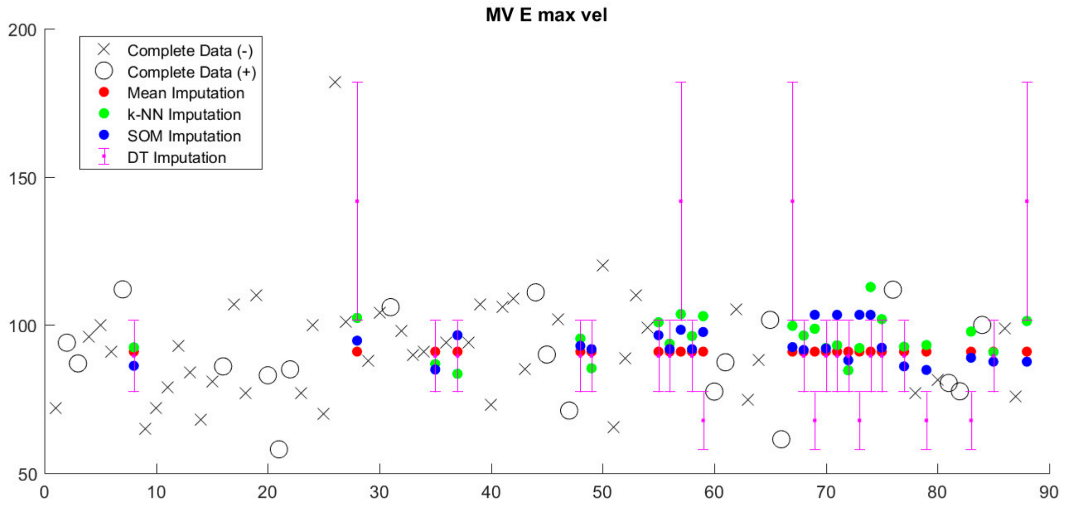
MV E max vel
Complete Data (-)
Complete Data (+)
Mean Imputation
k-NN Imputation
SOM Imputation
DT Imputation

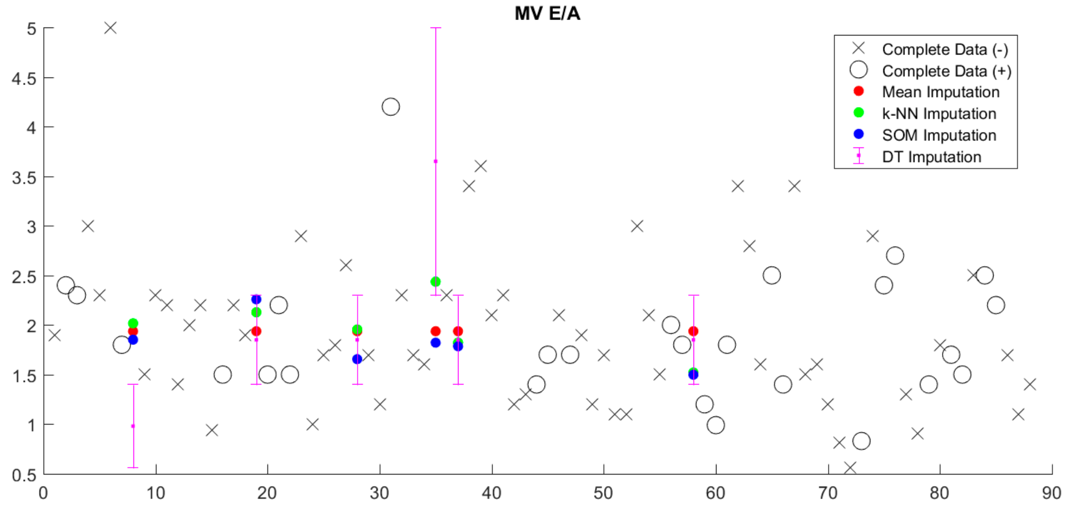
MV E/A
Complete Data (-)
Complete Data (+)
Mean Imputation
k-NN Imputation
SOM Imputation
DT Imputation

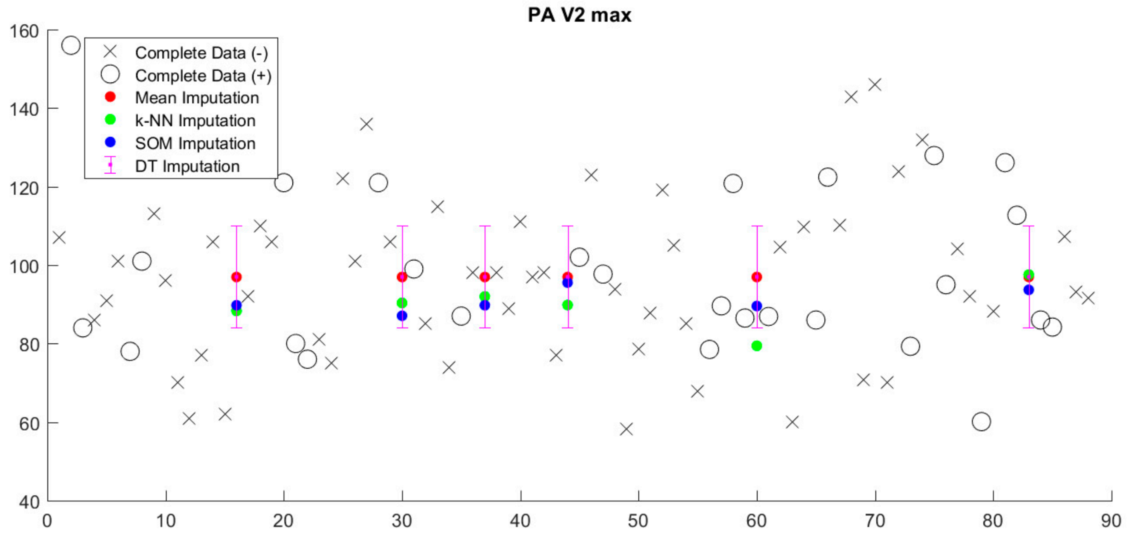
PA V2 max
Complete Data (-)
Complete Data (+)
Mean Imputation
k-NN Imputation
SOM Imputation
DT Imputation
160
140
120
100
80
60
40
0
10
20
30
40
50
60
70
80
90

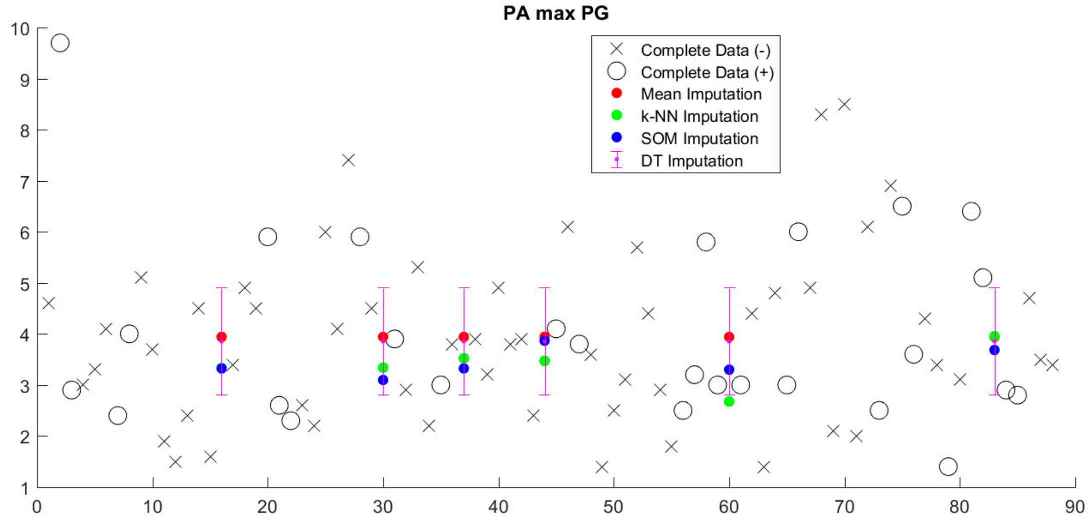
PA max PG
Complete Data (-)
Complete Data (+)
Mean Imputation
k-NN Imputation
SOM Imputation
DT Imputation
10
9
8
7
6
5
4
3
2
1
0
10
20
30
40
50
60
70
80
90

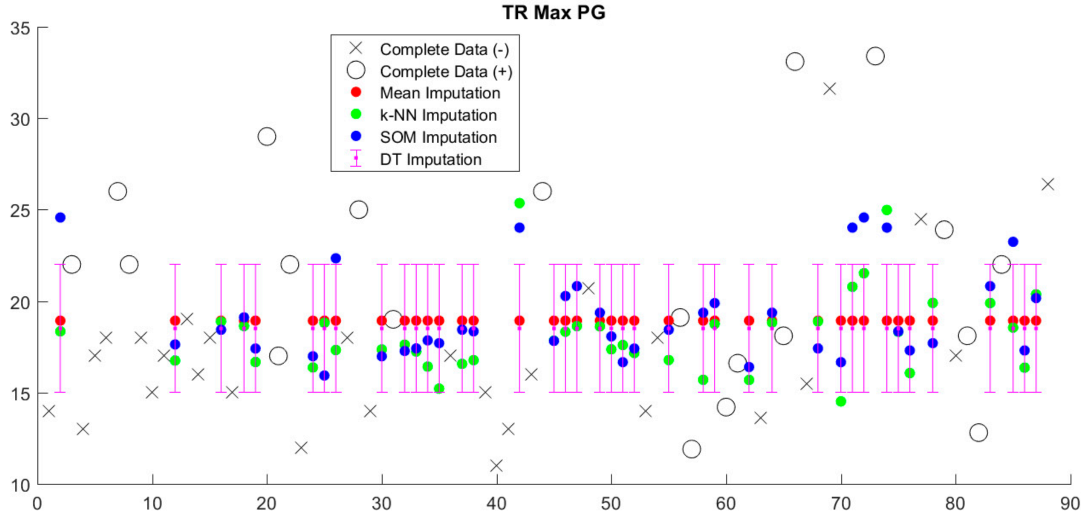
TR Max PG
Complete Data (-)
Complete Data (+)
Mean Imputation
k-NN Imputation
SOM Imputation
DT Imputation
35
30
25
20
15
10
0
10
20
30
40
50
60
70
80
90

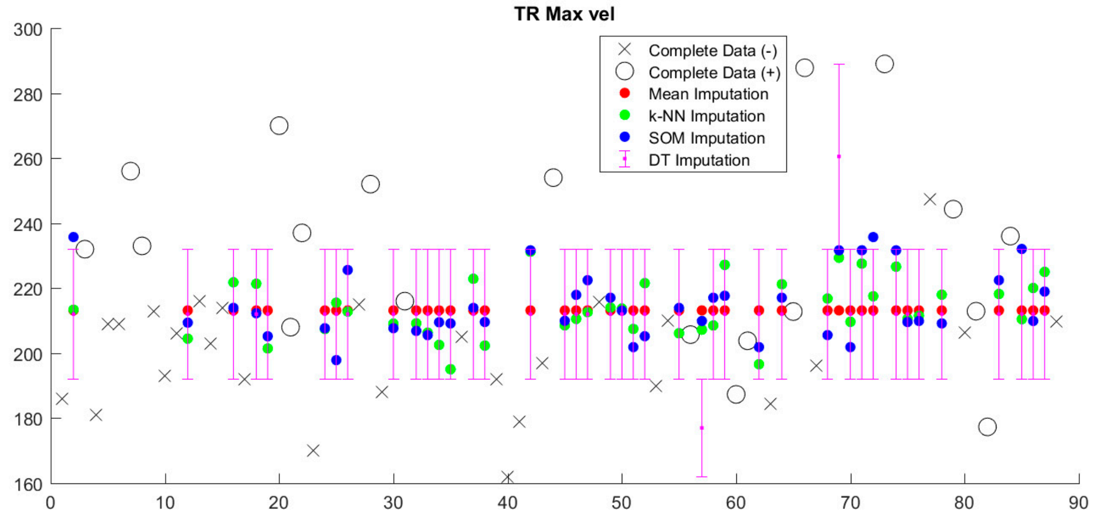
TR Max vel
Complete Data (-)
Complete Data (+)
Mean Imputation
k-NN Imputation
SOM Imputation
DT Imputation

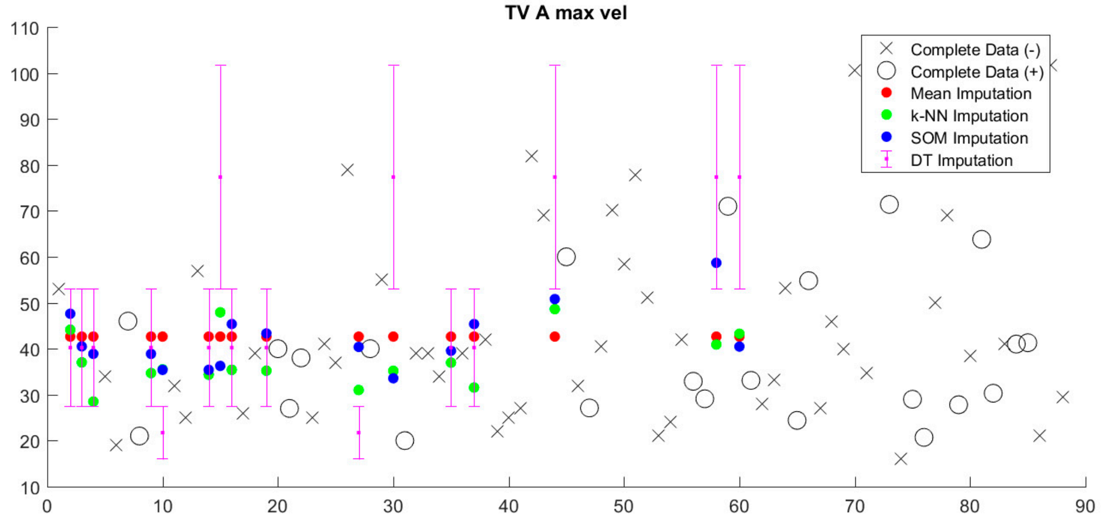
TV A max vel
Complete Data (-)
Complete Data (+)
Mean Imputation
k-NN Imputation
SOM Imputation
DT Imputation

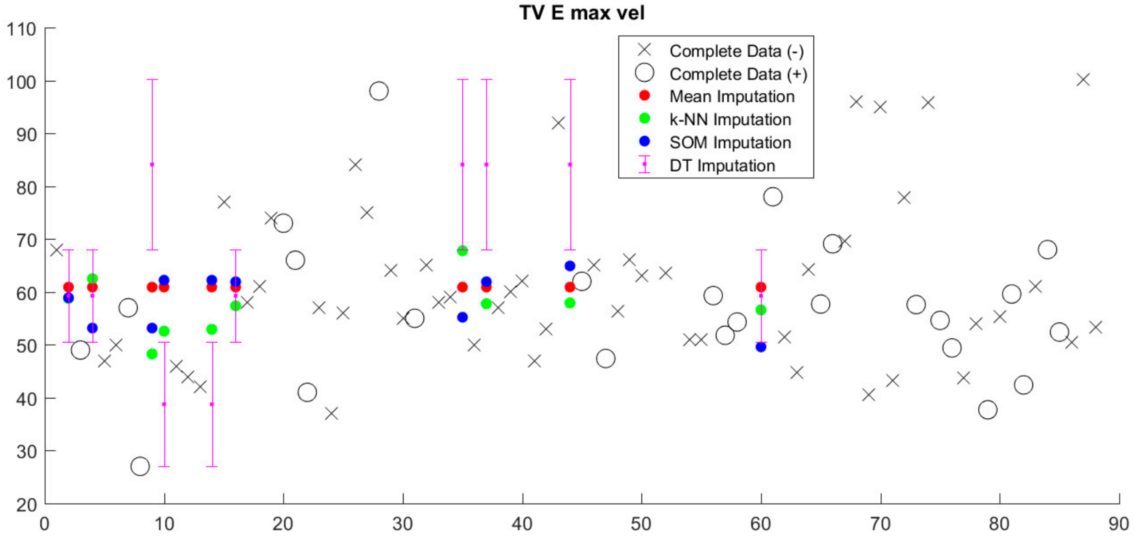
TV E max vel
Complete Data (-)
Complete Data (+)
Mean Imputation
k-NN Imputation
SOM Imputation
DT Imputation

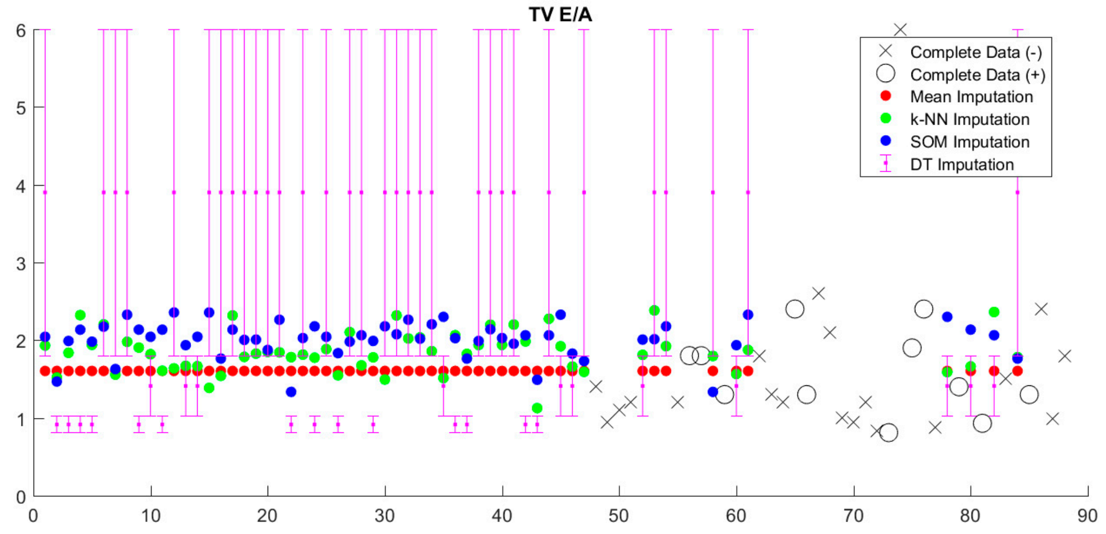
TV E/A
Complete Data (-)
Complete Data (+)
Mean Imputation
k-NN Imputation
SOM Imputation
DT Imputation

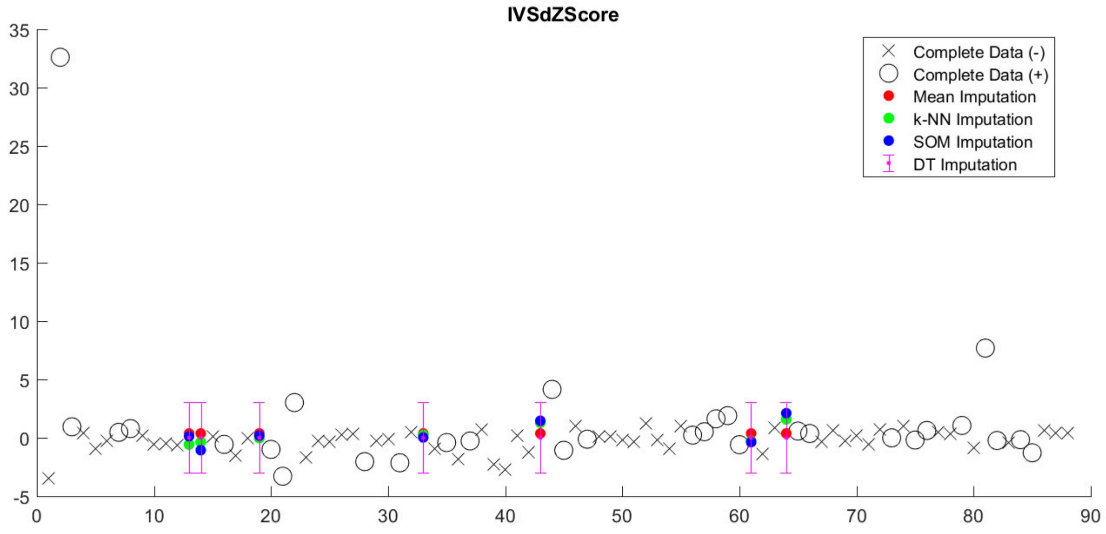
IVSdZScore
Complete Data (-)
Complete Data (+)
Mean Imputation
k-NN Imputation
SOM Imputation
DT Imputation

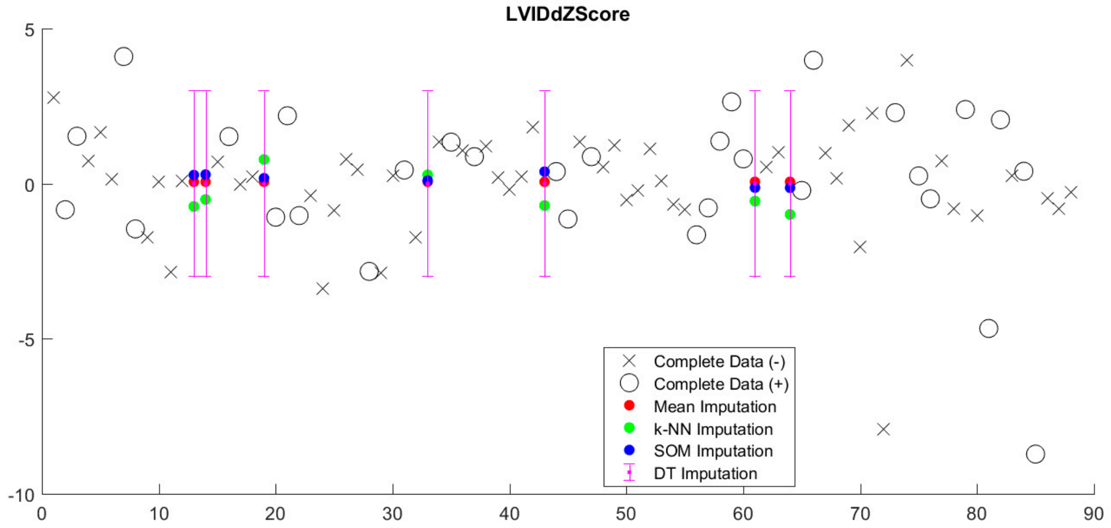
LVIDdZScore
Complete Data (-)
Complete Data (+)
Mean Imputation
k-NN Imputation
SOM Imputation
DT Imputation

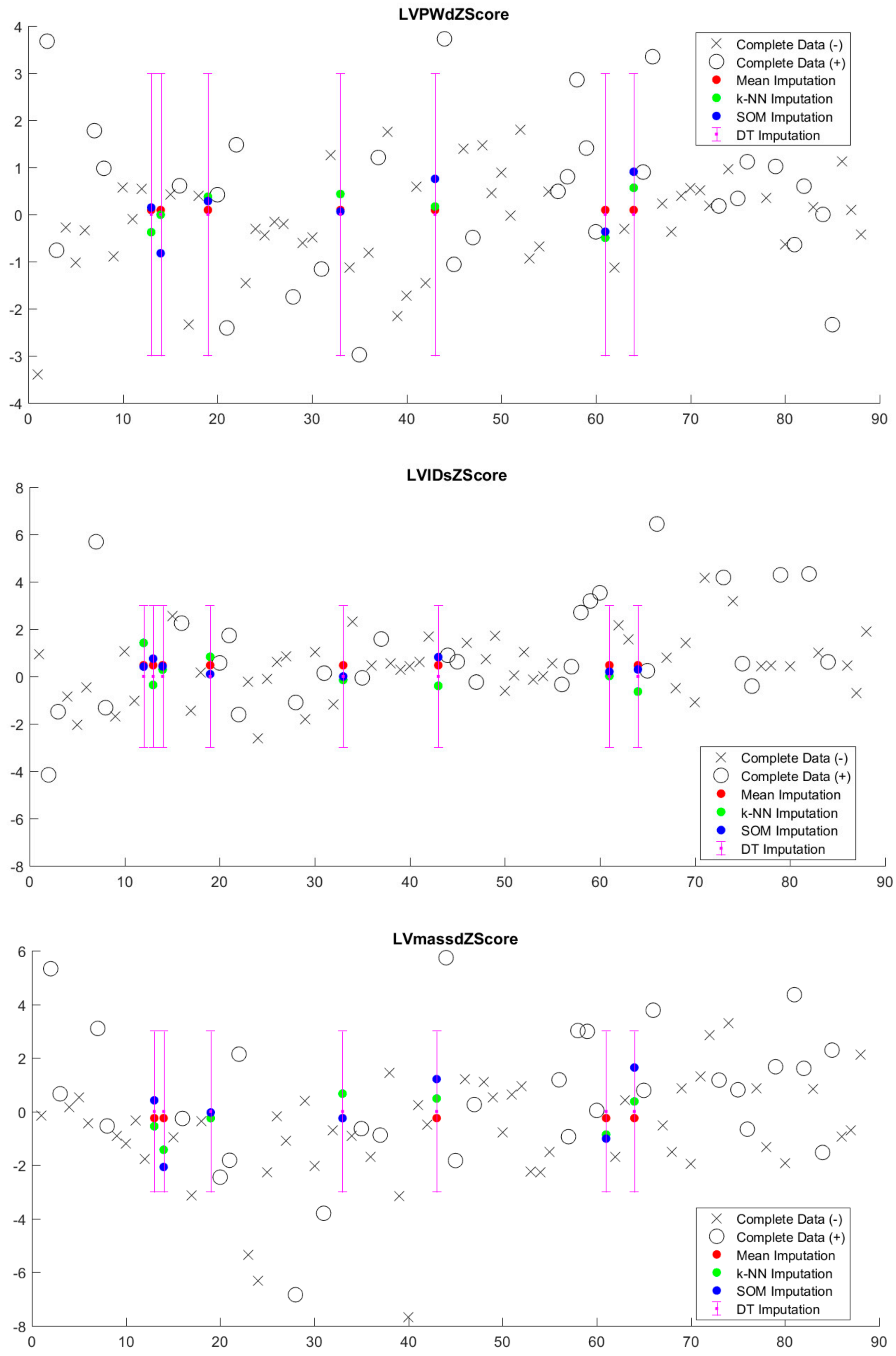

Figure S1. Imputed values for the variables in the 27-variable dataset.

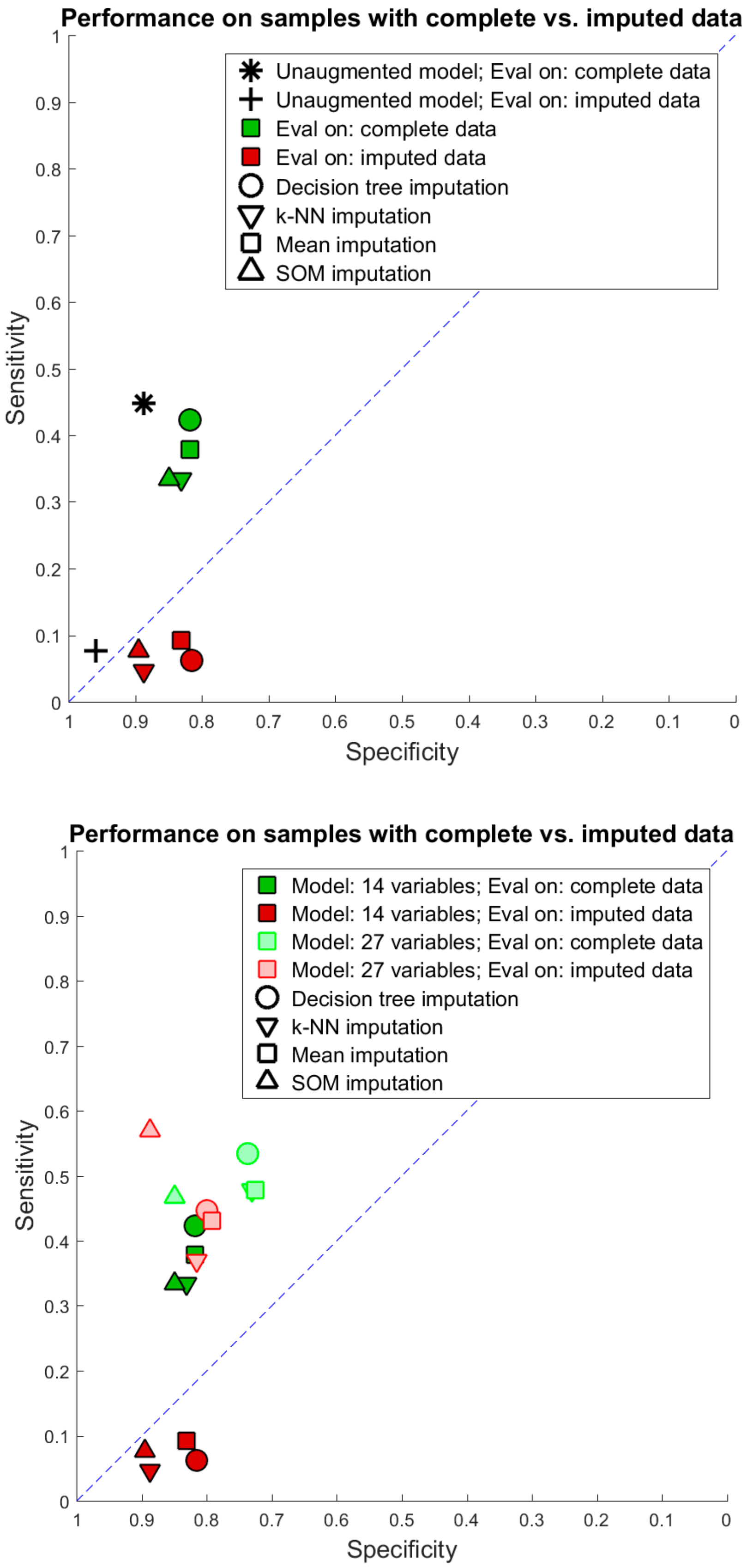

Figure S2. Performance of 14 and 27-variable imputation-augmented rulesets when values were discretized pre-imputation using a weighted frequency binning method.

Table S1. Review of papers comparing performance of imputation methods.

Paper	Imputation Methods	Dataset Types
Kang (2013) [1]	Locally linear reconstruction (LLR) vs. mean imputation, hot deck, k-NN, expectation conditional maximization, k-means, and mixture of Gaussians	13 real world datasets with simulated missingness 1%–50% (MCAR)
	**Evaluation Metric**	**Performance Finding**
	Accuracy (%) improvement compared to listwise deletion when training several classifiers	Neural network classifier:<br>At 1% missing values: Worst performing was +0.39% and best was +0.86%.<br>At 50% missing values: Worst performing was +36.77% and best was +44.09%.<br>Other classifiers similar.<br>LLR performed best in majority of tests, but not by much.
Batista and Monard (2003) [2]	k-NN, mean/mode imputation, and no imputation	4 real world datasets with simulated missingness 0%–50% (MCAR)
	Error rates when training decision tree classifiers	Pima dataset, C4.5, 1 attribute missing:<br>At 10% missing values: 26.17% with no imputation, 26.42% with mean imputation, 24.86% with k-NN imputation.<br>Performance varied with dataset.<br>No imputation performed better than k-NN imputation in 11/108 comparisons.<br>Mean imputation performed better than k-NN imputation in 20/108 comparisons.
Troyanskaya et al. (2001) [3]	k-NN vs. mean imputation and SVD imputation	Gene expression datasets with real missing values removed and simulated missing values added 1%–20% (MCAR)
	Normalized root mean squared error of imputed values versus original values	>0.40 for mean imputation, <0.33 for SVD imputation, and <0.25 for k-NN imputation.<br>SVD better than k-NN in cases where expression data is dominated by strong patterns. Both relatively insensitive to percentage of data missing.
Suyundikov et al. (2015) [4]	Weighted k-NN vs. MCMC and EM multiple imputation	Simulated gene expression datasets with simulated missing values added 10%–50% (MCAR)
	Root mean squared error of imputed values versus original values	MCMC and EM multiple imputation had RMSE around 1.5–2.0 for various missing percentages and sizes of datasets, while weighted k-NN had RMSE around 1.3.
Lakshminarayan et al. (1996) [5]	C4.5 decision tree imputation vs. Autoclass	Real world dataset with missing values (assumed MCAR)
	Error rate of classification task	C4.5 decision trees achieved an error rate of 22.6% compared to Autoclass, which achieved an error rate of 48.7%.
Twala (2009) [6]	Decision trees, mean/mode imputation, EM single and multiple imputation (EMSI and EMMI), listwise deletion (LD)	Real world datasets with simulated missing values (MCAR, MAR, and MNAR)
	Excess error % compared to performance on complete dataset using decision tree classification	On average, around 5% excess error difference between worst performing (LD) and best performing (EMMI), with slightly higher rates and differences in MNAR compared to MAR

		compared to MCAR. DT performed within 3% points of LD and EMMI.
Fessant and Midenet (2002) [7]	Self-organizing map (SOM) vs. mean imputation, hot deck, and multi-layered perceptron (MLP)	Real world dataset with simulated missing values (MCAR)
	Mean squared error of continuous variables, error percentages of categorical variables	MLP performed the best in continuous variables, and SOM performed best in categorical variables. Performance of SOM, MLP, and hot deck were very similar for all variables, and mean imputation performed worse for 3 of 4 variables imputed.
Vatanen et al. (2015) [8]	SOM, Imputation SOM, generative topographic mapping (GTM), Variational Bayesian principal components analysis (VBPCA), and mean imputation	Real world datasets with simulated missing values 10%–50% (MCAR)
	Root mean squared error of imputed values to original values	Both SOM/GTM methods and VBPCA beat mean imputation. SOM/GTM methods slightly underperformed compared to VBPCA on 10%–40% missing data, and performed equally to VBPCA on 50% missing data. Mean imputation achieved around 1.01 RMSE, VBPCA from 0.71 to 0.82, SOM from 0.74 to 0.83, Imputation SOM from 0.75 to 0.82, and GTM from 0.75 to 0.82.
Di Zio et al. (2004) [9]	Bayesian networks, hot deck	Real world dataset with simulated missing values 5%–10% (MCAR and MAR)
	Δ, a sum of differences in frequency of each category of a variable in a dataset	Bayesian networks established a Δ of ~0.25 in MAR and ~0.11 in MCAR. Hot deck methods achieved a Δ of 0.24–0.36 in MAR and 0.90–0.13 in MCAR.<br>On average, the Δ for Bayesian networks was about 73% that of hot decking in MAR and about 85%–92% in MCAR.
Hruschka et al. (2007) [10]	Bayesian networks, EM, MCMC data augmentation, decision trees, and mean/mode imputation	Real world datasets with simulated missing values (MCAR)
	Distances to original of continuous variables and error percentages of categorical variables; average correct classification rate of imputed data in classification tasks	Bayesian networks performed similarly to decision trees in imputation error. Performance on the classification task was highly variable between datasets for each method.
Jerez et al. (2010) [11]	Listwise deletion, mean imputation, hot deck, multiple imputation (using SAS, Amelia, and MICE), MLP, k-NN, and SOM	Real world dataset with real missing values of around 5.61% (assumed MAR)
	AUC of neural network classification task	LD: 0.715<br>Mean: 0.723<br>Hot deck: 0.711<br>SAS: 0.722<br>Amelia: 0.717<br>MICE: 0.725<br>MLP: 0.734<br>k-NN: 0.735<br>SOM: 0.733<br>Standard devations of AUC were around 0.03 for all. Machine learning methods (MLP, k-NN,

		SOM) produced statistically significant differences compared to LD.
Rahman and Davis (2013) [12]	Fuzzy unordered rule induction algorithm (FURIA) vs. decision tree, SVM, k-NN, and mean imputation	Real world dataset with real missing values, with variables containing 1%–30% missing values and cases containing 4%–56% missing values (assumed MAR)
	Accuracy of subsequent classification task	With decision tree classification:<br>Decision tree imputation: 0.8<br>k-NN: 0.8<br>FURIA: 0.8<br>SVM: 0.78<br>Mean: 0.8<br>With k-NN classification:<br>Decision tree: 0.71<br>k-NN: 0.81<br>FURIA: 0.79<br>SVM: 0.71<br>Mean: 0.77
de Souto et al. (2015) [13]	Mean imputation, median imputation, k-NN, Bayesian PCA, linear least squares	Real world dataset with real missing values (from 0.76% to 3.34% missing after filtering out variables with >10% missing values) (assumed MCAR)
	Classification error	No statistically significant difference found between imputation methods over a range of classification methods.

References

1. Kang, P. Locally linear reconstruction based missing value imputation for supervised learning. *Neurocomputing* **2013**, *118*, 65–78.
2. Batista, G.E.A.P.A.; Monard, M.C. An analysis of four missing data treatment methods for supervised learning. *Appl. Artif. Intell.* **2003**, *17*, 519–533.
3. Troyanskaya, O.; Cantor, M.; Sherlock, G.; Brown, P.; Hastie, T.; Tibshirani, R.; Botstein, D.; Altman, R.B. Missing value estimation methods for DNA microarrays. *Bioinformatics* **2001**, *17*, 520–525.
4. Suyundikov, A.; Stevens, J.R.; Corcoran, C.; Herrick, J.; Wolff, R.K.; Slattery, M.L. Accounting for dependence induced by weighted KNN imputation in paired samples, motivated by a colorectal cancer study. *PLoS ONE* **2015**, *10*, e0119876.
5. Lakshminarayan, K.; Harp, S.A.; Goldman, R.P.; Samad, T. Imputation of Missing Data Using Machine Learning Techniques. In The Second International Conference on Knowledge Discovery and Data Mining (KDD-96), Portland, OR, USA, 2–4 August 1996; pp. 140–145.
6. Twala, B. An Empirical Comparison Of Techniques For Handling Incomplete Data Using Decision Trees. *Appl. Artif. Intell.* **2009**, *23*, 373–405.
7. Fessant, F.; Midenet, S. Self-Organising Map for Data Imputation and Correction in Surveys. *Neural. Comput.* **2002**, *10*, 300–310.
8. Vatanen, T.; Osmala, M.; Raiko, T.; Lagus, K.; Sysi-Aho, M.; Orešič, M.; Honkela, T.; Lähdesmäki, H. Self-organization and missing values in SOM and GTM. *Neurocomputing* **2015**, *147*, 60–70.
9. Di Zio, M.; Scanu, M.; Coppola, L.; Luzi, O.; Ponti, A. Bayesian networks for imputation. *J. R. Stat. Soc. Ser. A (Statistics Soc.)* **2004**, *167*, 309–322.
10. Hruschka, E.R.; Hruschka, E.R.; Ebecken, N.F.F. Bayesian networks for imputation in classification problems. *J. Intell. Inf. Syst.* **2007**, *29*, 231–252.
11. Jerez, J.M.; Molina, I.; García-Laencina, P.J.;Alba, E.; Ribelles, N.; Martín, M.; Franco, L. Missing data imputation using statistical and machine learning methods in a real breast cancer problem. *Artif. Intell. Med.* **2010**, *50*, 105–115.

12. Rahman, M.M.; Davis, D.N. Machine Learning-Based Missing Value Imputation Method for Clinical Datasets. In *IAENG Transactions on Engineering Technologies: Special Volume of the World Congress on Engineering 2012*; Yang, G.-C., Ao, S., Gelman, L., Eds.; Springer: Dordrecht, The Netherlands, 2013; pp. 245–257.
13. De Souto, M.C.P.; Jaskowiak, P.A.; Costa, I.G. Impact of missing data imputation methods on gene expression clustering and classification. *BMC Bioinform.* **2015**, *16*, 64.
